# Ablation of LAT2 Transporter Causes Intramuscular Glutamine Accumulation and Inhibition of Fasting‐Induced Proteolysis

**DOI:** 10.1002/jcsm.13847

**Published:** 2025-06-23

**Authors:** Meritxell Espino‐Guarch, Susie Shih Yin Huang, Clara Vilches, Esther Prat, Rana El Nahas, Ghalia Missous, Susanna Bodoy, Abbirami Sathappan, Mohammad Ameen Al‐Aghbar, Clara Mayayo, Montse Olivé, Silvia Busquets‐Rius, David Sebastián, Antonio Zorzano, Manuel Palacin, Nicholas van Panhuys, Virginia Nunes

**Affiliations:** ^1^ Laboratory of Immunoregulation, Research Department, Sidra Medicine Doha Qatar; ^2^ Washington University School of Medicine St. Louis USA; ^3^ Genes, Disease and Therapy Program, Human Molecular Genetics Laboratory – Institut Investigació Biomèdica de Bellvitge (IDIBELL) L’Hospitalet de Llobregat Barcelona Spain; ^4^ Institut de Ciències Fotòniques (ICFO) The Barcelona Institute of Science and Technology Barcelona Spain; ^5^ Institute for Research in Biomedicine (IRB Barcelona) The Barcelona Institute of Science and Technology (BIST) Barcelona Spain; ^6^ Department of Biochemistry and Molecular Biomedicine, Universitat de Barcelona Barcelona Spain; ^7^ U731, Centro de Investigación Biomedica En red de Enfermedades Raras (CIBERER) Barcelona Spain; ^8^ Advanced Imaging Core Facility Sidra Medicine Doha Qatar; ^9^ Neuromuscular Disorders Unit, Neurology Department Hospital de la Santa Creu i Sant Pau Barcelona Spain; ^10^ Centro de Investigación Biomédica en Red en Enfermedades Raras (CIBERER) Spain; ^11^ Cancer Research Group, Departament de Bioquimica I Biomedicina Molecular, Facultat de Biologia Universitat de Barcelona Barcelona Spain; ^12^ Department of Biochemistry and Physiology, School of Pharmacy University of Barcelona Barcelona Spain; ^13^ Centro de Investigación Biomédica en Red de Diabetes y Enfermedades Metabólicas Asociadas (CIBERDEM); ^14^ Institute for Research in Biomedicine (IRB Barcelona) The Barcelona Institute of Science and Technology Barcelona Spain; ^15^ College of Health and Life Sciences Hamad Bin Khalifa University Doha Qatar

**Keywords:** ageing, glutamine, LAT2, mTORC1, proteolysis, skeletal muscle

## Abstract

**Background:**

The neutral amino acid transporter *SLC7A8* (LAT2) has been described as a key regulator of metabolic adaptation. LAT2 mutations in human populations have been linked to the early onset of age‐related hearing loss and cataract growth. As LAT2 was previously found to be highly expressed in skeletal muscle, here we characterised its role in the regulation of *skeletal muscle* amino acid flux and metabolic adaptation to fasting.

**Methods:**

Wild‐type (WT) and LAT2 knock‐out (LAT2KO) mice were exposed to short‐ and long‐periods of fasting (16 and 48 h). The impact of the absence of LAT2 on amino acid content, gene expression, proteolysis activity, muscle tone, and histology was measured. To characterise the impact on muscle degradation, we tested LAT2 KO mice in cancer‐associated cachexia, streptozocin‐induced Type‐1 diabetes, and ageing models.

**Results:**

LAT2KO mice experienced a notable reduction in body weight during fasting (WT:14% and LAT2KO:18%, *p* = 0.02), with a greater reduction in fat mass (0.5‐fold, *p* = 0.013) and a higher relative retention of muscle mass (1.3‐fold, *p* = 0.0003) compared with WT. The absence of LAT2 led to increased intramuscular glutamine (Gln) accumulation (6.3‐fold, *p* < 0.0001), accompanied by a reduction in skeletal muscle proteolysis during fasting (0.61‐fold, *p* = 0.0004) primarily due to decreased proteasomal and autophagic activity (0.45‐fold, *p* = 0.016 and 0.7‐fold, *p* = 0.002, respectively). Ex vivo incubation of LAT2KO muscle with rapamycin recovered proteolysis function, demonstrating a mTORC1‐dependent pathway. Decreased proteolysis in LAT2KO animals was associated with increased mTORC1 translocation to the lysosome (mTORC1‐Lamp1 colocalization in fasted LAT2KO muscles was 1.23‐fold, *p* < 0.0001). Of the three muscle loss models tested, differences were observed only during ageing. Young LAT2KO mice (3 M) exhibited muscle tone and MurF1 expression levels comparable to those of older WT mice (12 M) (0.44‐fold, *p* = 0.02 and 0.48‐fold, *p* = 0.04, respectively).

**Conclusion:**

LAT2 has a critical role in regulating Gln efflux from skeletal muscle. The absence of LAT2 led to elevated intracellular Gln levels, impairing muscle proteolysis by inducing mTORC1 recruitment to the lysosome. Further, chronic Gln accumulation and decreased proteolysis were found to induce the early onset of an age‐related muscle phenotype.

## 1 Introduction

Skeletal muscle, which constitutes 40% of the total human body weight, is a central component of metabolism, serving as the primary reservoir of amino acids (AAs) during periods of fasting. The degradation of muscle tissue under fasting conditions is a complex process influenced by factors such as fasting duration, fat reserves, and metabolic activity [1]. The ubiquitin–proteasome system (UPS) and the autophagy–lysosome pathway are the two main protein degradation systems involved in muscle catabolism. The UPS coordinates the targeted degradation of proteins, which is regulated through mTORC1‐ULK1 signalling. In comparison, autophagy breaks down cellular components for recycling and is regulated by the mTORC1 and AMPK pathways [2]. Autophagy and UPS are tightly controlled by a complex system of regulatory crosstalk, with reciprocal communication occurring between the two pathways. As proteasomal activation delays autophagosome fusion with lysosomes, the UPS system acts as an on/off switch for the autophagy process [3].

Excessive muscle wasting due to increased proteolysis, often accompanied by reduced protein synthesis, plays a role in a diverse array of pathological conditions, ranging from natural ageing to chronic illnesses such as cancer, metabolic disorders, inflammatory diseases (e.g., rheumatoid arthritis), infectious diseases (e.g., acquired immunodeficiency syndrome), and sepsis [4].

AAs serve not only as building blocks for proteins but also as direct regulators of the metabolic pathways involved in protein turnover. Leucine, arginine (Arg), and phenylalanine, amongst others, exert their signalling effects through mTORC1 [5]. The upstream regulation of mTORC1 can also be controlled by both the cytoplasmic and lysosomal AA content. Moreover, mTORC1 is activated through direct phosphorylation when it is translocated to the lysosomal surface through the amino‐acid‐sensitive complex [6]. Additionally, AA sensors such as the sestrin family of proteins directly regulate mTORC1 signalling [7, 8]. Whereas under the conditions of AA deprivation, AMPK is activated to suppress mTORC1 activity and enhance autophagy [9, 10].

Solute Carrier Family 7 Member 8 (LAT2 or *SLC7A8*) is the catalytic subunit 2 of the Na^+^‐independent transporter employed in the System L transport of large and aromatic neutral AAs [11, 12]. LAT2/4F2hc (*SLC7A8/SLC3A2*) transports a wide variety of neutral AAs, with the exception of proline, which is a poor substrate for the alternate exchanger LAT1 (*SLC7A5*) [13]. LAT2, unlike LAT1, mediates an efficient efflux of small molecules, including alanine, cystine, serine (Ser), and Gln. The nonessential AA Gln, transported by LAT2, is essential for tissue homeostasis, energy production, and cellular growth, particularly during nutrient deprivation [13]. During periods of nutrient deprivation, Gln plays critical roles by providing energy in the form of ATP and in the biosynthesis of glutathione, an antioxidant that helps to maintain the cellular redox state [13]. Furthermore, it has been demonstrated that under in vitro conditions, Gln plays a crucial role in inhibiting cellular autophagy via mTORC1‐S6K1 [14].

Increased LAT2‐mediated AA uptake is associated with poor overall survival in different types of cancer. High LAT2 expression in osteosarcoma [15] and pancreatic cancer cells [16] decreases antitumour immune response and promotes chemoresistance by Gln‐dependent mTORC1 activation. Additionally, in innate lymphoid cells Type 2 (ILC2) LAT2‐mediated transport has been described as a key regulator of metabolic adaptation [17]. Considering these studies suggesting that LAT2 plays a pivotal role in the regulation of AA transport, particularly during periods of metabolic adaptation, together with the observation that LAT2 is highly expressed in skeletal muscle, and under fasting conditions, its expression increases up to 6‐fold; here, we sought to elucidate the role of the LAT2 transporter in regulating Gln efflux from murine skeletal muscle and how the lack of LAT2 alters metabolic responses to fasting.

## 2 Methods

### 2.1 In Vivo Model Systems

LAT2KO mice were generated as described in [18]. Mice housed in groups of 4–5 with chow diet ad libitum and fasted either overnight (16 h) or for 48 h with only water access according to the approved protocol in Idibell Animal Facility (Spain). Measurements of autophagic flux by western blot were conducted following an intraperitoneal injection of chloroquine (100 mg/kg) to wild type (WT) and LAT2 knockout (KO) mice 2 h prior to dissection [19]. Protein synthesis was measured by SUnSET protocol injecting puromycin 16 h prior to dissection as described in [20]. Protein was extracted from muscles, and a western blot was performed. A grip‐strength test was performed for the forelimbs by pulling each mouse’s tail back whilst ensuring it maintained grip in the grid, with measurements repeated five times per animal. A treadmill assessment was conducted that involved increasing the treadmill speed. From a starting speed of 14 cm/s, it was increased after 20 min and then progressively every 2 min to 32 cm/s. An initial acclimation for 15 min daily for 2–3 days was carried out prior to the assessments. A Type 1 diabetes (T1D) model was induced in WT and LAT2KO mice by administration of streptozotocin (STZ), a compound with pancreatic β‐cell toxicity, at a single high dose of 200 mg/kg. Then, 7–10 days post‐STZ injection, blood glucose levels were evaluated to determine the induction of the diabetic phenotype. A cancer cachexia (CA) model was induced by injection of 1 × 10^6^ Lewis lung carcinoma cells or saline into the hind flanks of WT and LAT2KO mice [21]. Body weight, tumour volume, and food intake were monitored every week for 3 months after the inoculations.

### 2.2 Proteolysis Ex Vivo

Extensor digitorum longus (EDL) muscles were incubated in Krebs–Henseleit–Hepes buffer with 25 mM of cycloheximide, and the muscles were studied at 35°C and gassed with 95% O_2_ and 5% CO_2_ for 2 h with or without inhibitors. Muscles were then weighed and frozen for AA quantification. An aliquot of 2 mL incubation media was used for tyrosine quantification by fluorometric absorbance (Ex: 460 nm, Em: 570 nm) in a SpectraMax Spectrofluorometer (Molecular Devices).

### 2.3 Measurement of AA Content

Plasma or tissue powder was deproteinized, and an internal standard was added (2000 μM norLeucine). Quantitative analysis of AAs was performed by chromatographic separation by cation‐exchange chromatography and followed by post‐column derivatization with ninhydrin and UV detection [22]. AA peaks were identified according to the retention times of the corresponding standards.

### 2.4 Biochemistry and Enzyme Activity

Plasma and urine were collected to measure several parameters by colorimetric commercial assays following the manufacturer’s instructions. A free fatty acid (FFA) quantitation kit (MAK0044), ketone body (KB) assay (MAK134), urea assay kit (MAK006), ammonia assay kit (MAK538) and ALT assay kit (MAK052) were all obtained from Sigma‐Aldrich. Muscle extracts were used to measure proteasome activity using the following Luminescent Promega Assays: Proteasome‐Glo Chymotrypsin‐Like Cell‐Based Assay (G8660), Proteasome‐Glo Trypsin‐like cell‐Based Assay (G8760) and Cell‐Based Proteasome‐Glo Assay Caspase like (G8860).

### 2.5 Real‐Time PCR and RNA‐Seq

RNA from G + S muscles was extracted using Trizol, and cDNA was synthesised using 1 μg of RNA and a First Strand cDNA Synthesis kit (Roche). Quantitative PCR was performed on the LightCycler 480 System (Roche) using a Universal Probe Library and 384‐well plates. Primers and probes list in Table S5A. For RNA library preparation, the TruSeq Stranded RNA Illumina kit was used, and the library was sequenced in a HiSeq4000 system at Sidra Medicine OMICs Facility. Alignments of reads in .BAM file format were performed in START. RNA‐Seq reads from G + S muscles (*N* = 3–5 per genotype and intervention) were aligned to the mm10 mouse genome and mapped to GRCm38.p6.

### 2.6 Western Blot

Total protein was extracted using 1% Triton 100X containing phosphatase and protease inhibitors. Total membranes were extracted by ultracentrifugation gradient in a Hepes–EDTA–sucrose buffer containing protease inhibitors. Protein content was quantified by Pierce BCA Protein Assay kit (Thermo Scientific). Acrylamide gels and PSVF membranes were used for western blotting. After primary and secondary antibody, see incubation and dilution details in Table S5B, proteins were detected by enhanced chemiluminescent reaction Pierce ECL (Thermo Scientific) and autoradiography or using fluorescence and the Odyssey instrument (LI‐COR).

### 2.7 Histology

We stained 10‐μm thick cryostat sections with haematoxylin and eosin, modified Gömori trichrome, periodic acid Schiff technique, reduced nicotinamide adenine dinucleotide dehydrogenase–tetrazolium reductase, succinic dehydrogenase and cytochrome c oxidase as described [23].

### 2.8 Immunofluorescence

Formalin‐fixed paraffin–embedded sections (5 μm) were deparaffinised and submitted to antigen retrieval by microwaving at 95°C for 10 min at 80% power in citrate buffer, pH 6 (#C9999, Sigma). Following blocking and permeabilization (in 1% BSA, 1% goat serum, and 0.05% Tween20, TBST), primary and secondary antibody incubations were performed as indicated in Table S5B. Subsequent sections were mounted using Vectashield H‐1000 with DAPI (H‐1200‐10, Vector Laboratories). High‐resolution imaging was achieved with an LSM 880 confocal microscope (Carl Zeiss, Germany) and employing Airyscan superresolution detectors. Images were captured using both 20x and 63x oil immersion objectives and processed using Imaris Software 9.0 (Bitplane).

### 2.9 Mitochondrial Function and Quantification

Saponin‐permeabilized muscle fibre bundles were microdissected from WT and LAT2KO gastrocnemius muscle and loaded onto a high‐resolution respirometer (Oroboros Oxygraph‐2k). Titrations and oxygen measurements were carried out in 2 mL of Mi05 medium and normalised to sample weight. qRT‐PCR using 4 mitochondrial genes and 2 genomic genes was done using DNA extracted from gastrocnemius muscle. To determine mitochondrial load, mitochondrial gene expression (tRNA, Nd2 and Nd4) was normalised by genomic genes (PPia) (2^DCt^ where DCt is Ct _genomic_‐Ct _mitochondria_. Primers sequence 5′–3′: tRNA(F: CCCAGCTACTACCATCATTCAAGT+R: GATGGTTTGGGAGATTGGTTGATGT), Nd2 (F: ATCCTCCTGGCCATCGTACT+R: ATCAGAAGTGGAATGGGGCG), Nd4 (F: AGCTCAATCTGCTTACGCCA+R: TGTGAGGCCATGTGCGATTA) and PPia (F: TGCTGTGTCGGTAGCCATTT+R: CCAAAGACCACATGCTTGCC). Pdk2 was used as a genomic control (F: CTCGCCGTTTACCCATTCCT+R:TTCAGCAAGTTCTCCCCGTC).

### 2.10 Statistical and Analysis Tools

Data plots and statistics had been analysed using GraphPad Prism Software (Version 9). RNASeq raw counts were converted to cpm and analysed using iDEP2.0 integrated web platform [24]. Raw counts transformation for clustering and PCA was by EdgeR:log2(CPM + c) keeping only genes with minimal one count per million (CPM) in at least four samples. Differential expression analysis was performed using DESeq2. Genes with an adjusted *p* value < 0.05 and a fold‐change > 1.5 were considered significant. For downstream analysis, GO biological processes and KEGG databases were utilised for enrichment analysis.

## 3 Results

### 3.1 A Lack of LAT2 Triggers Intramuscular Gln and Ser Accumulation

**FIGURE 1:**
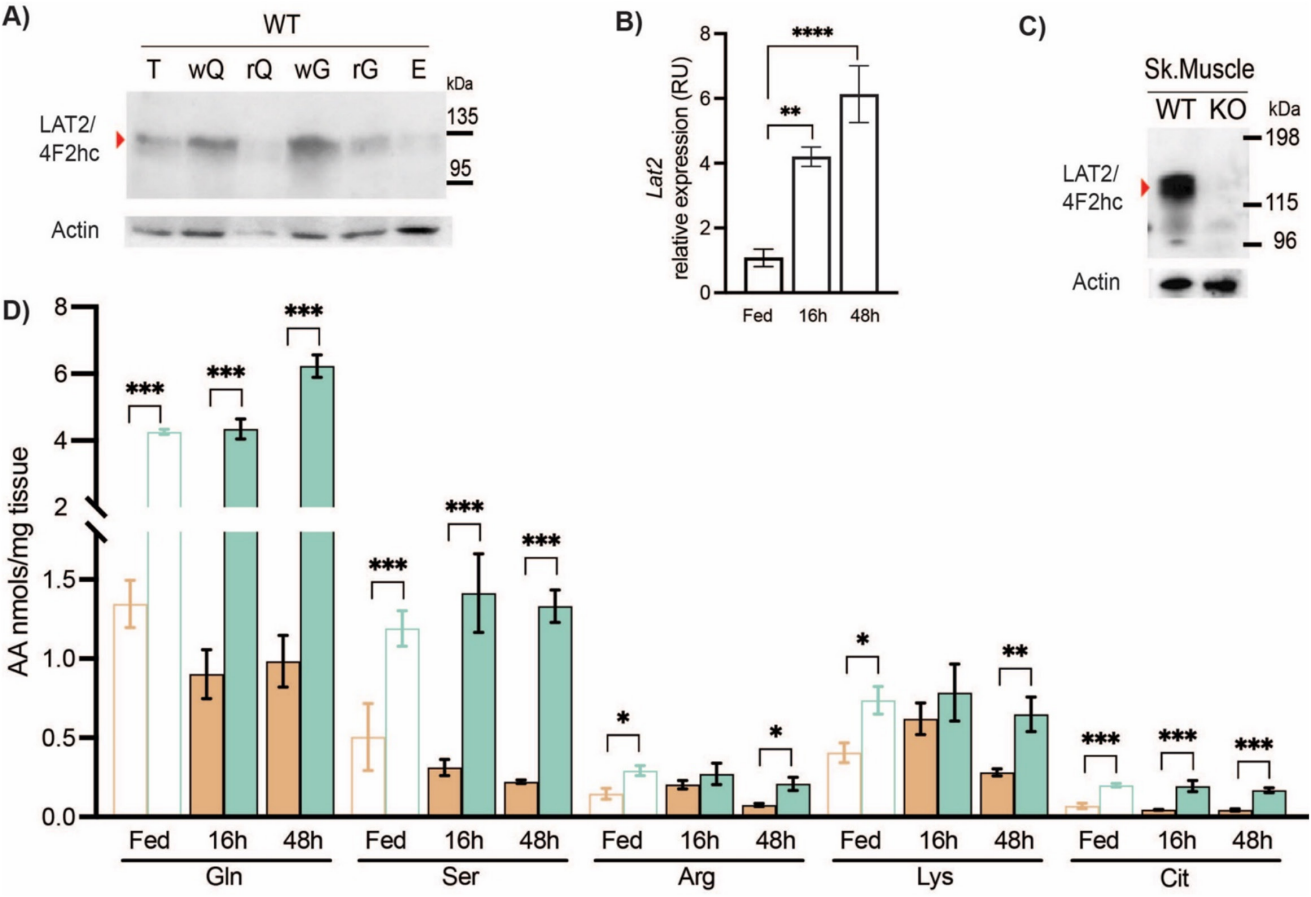
LAT2 expression in skeletal muscle and intramuscular AA content (A) Western blot of 50 μg of total skeletal muscle membranes from WT mice in non‐reducing conditions. T: tibialis, wQ: white quadriceps, rQ: red quadriceps, wG: white gastrocnemius, rG: red gastrocnemius + soleus, E: *Extensor digitorum longus*. (B) *Lat2* expression analysis by real time PCR of WT gastrocnemius sample from fed and 16‐ and 48‐h fasted mice, normalised to GAPDH expression (*N* = 6 per condition). (C) Western blot of 100 μg of total membranes of gastrocnemius (G) + soleus (S) muscle from WT and LAT2 KO mice in nonreducing conditions (red arrows indicate LAT2/4F2hc heterodimer ∼120 kDa). (D) AA content of G + S muscles at fed and 16‐ and 48‐h fasted mice per milligramme of tissue; *N* = 6–12 per condition and genotype, WT (orange), LAT2KO (turquoise). (B–D) Wilcoxon rank sum test; *p* values, * < 0.05, ** < 0.01, *** < 0.001 and **** < 0.0001.

Under homeostatic conditions, the LAT2 transporter was expressed in both white and red muscle fibres of WT mice (Figure 1A). Under catabolic conditions, in which the mice were fasted for either short (overnight, 16 h) or long (48 h) periods, the expression of *Lat2* increased 4‐ and 6‐fold, respectively, in gastrocnemius and quadriceps muscle compared to those in controls fed *ad libitum* (Fed) (Figure 1B). To assess the importance of LAT2 in the transport of AAs during fasting, we characterised *Slc7a8* ^−/−^ (LAT2KO) animals, in which the expression of LAT2 was absent (Figure 1C). Intramuscular AA content was analysed in mixed gastrocnemius and soleus (G + S) muscle (Figure 1D and Table S1), liver (Table S2) and plasma (Table S3) samples. Under Fed conditions, two neutral AAs, Gln and Ser, and two basic AAs, Arg and lysine (Lys), were present in significantly higher concentrations in LAT2KO muscles compared with WT group samples, with Gln being the most abundant AA present in G + S muscle (> 4 μmol/g) (Figure 1D). Following both 16‐ and 48‐h fasts, LAT2KO mice maintained their levels of Ser, Lys, and Arg, comparable to levels in the fed group, whereas these AAs were depleted in WT mice. Following fasting, significantly higher levels of Gln were found to be present in LAT2KO muscle at both time points analysed, at levels significantly higher (3‐ and 6‐fold, respectively) than those in WT muscle at the same time points (Figure 1D). Additionally, citrulline (Cit) was found to have accumulated in Fed LAT2KO muscles, with the levels maintained following 16 and 48 h of fasting, whereas a significant Cit decrease was observed in the fasted muscle of WT animals (Figure 1D and Table S1), liver (Table S2) and plasma (Table S3) samples.

The authors of previous studies primarily attributed muscle Gln transport to systems N and A [25]. In mouse skeletal muscle, we detected the presence of System A (*SLC38A1*, *SLC38A2* and *SLC38A4*), System N (*SLC38A3*), System y^+^L (*SLC7A6*), System ASC (*SLC7A10* and *SLC7A12*), and System L (*SLC7A5*, *SLC7A8*, *SLC43A1* and *SLC43A2*) transporters. Although amongst the transporters with a putative capacity to compensate for the absence of LAT2 activity, none were significantly upregulated in LAT2KO muscle compared to WT muscle (Table S4).

### 3.2 Absence of LAT2 Alters Proteostasis Pathways in Response to Fasting

**FIGURE 2:**
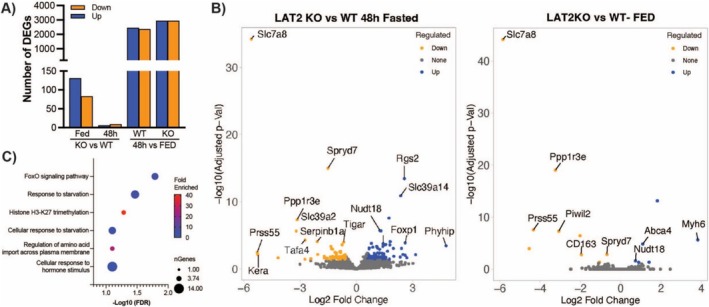
DEGs and enrichment analysis by RNA‐Seq analysis: (A) plot representing number of genes considered differentially expressed (DEGs) if they had minimum fold change ≥ 1.5 and FDR cutoff of < 0.05. (B) Volcano plot representing DEGs comparing LAT2KO versus WT at fed (fed) and 48 h fasting (left). (A, B) In blue significant up‐regulated and in orange significantly down‐regulated genes are represented. **(C)** Significant biological and KGEGG enrichment terms of 48‐h fasted DEGs comparing genotypes are represented. For each term significant differentially expressed genes is depicted by symbol size and fold enrichment by colour scale.

We next sought to characterise the metabolic impact of the AA imbalance observed in the absence of LAT2 under catabolic conditions on gene expression and performed RNA sequencing (RNA‐Seq) on WT and LAT2KO gastrocnemius muscle from fed and 48‐h fasted animals. We observed relatively few differentially expressed genes (DEGs) comparing genotypes with 15 and 214 DEGs present in fed and 48‐h fasted animals, respectively (Figure 2A,B). In comparison, large numbers of DEGs were identified following fasting with 4809 genes in WT and 5874 in the LAT2KO group (Figure 2A and Figure S1A). Notably, the effects of the fasting intervention (48 h vs Fed) were much more profound, contributing 55.6% to the coefficient of variance in comparison to genotype, which contributed 4% (Figure S1B,C). Enrichment analysis of DEGs comparing genotypes determined the presence of 6 GO biological terms and 1 KEGG module to be significantly associated following 48 h of fasting (Figure 2C). Alterations in AA import across plasma membrane regulation were 26‐fold enriched, and responses to starvation were found enriched over 5‐fold. Additionally, the FoxO signalling module was shown to be enriched by 6‐fold, respectively. Together, these results indicate that fasting has an outsized effect on regulating gene expression in muscle irrespective of genotype. However, the absence of LAT2 also led to significant changes in fundamental metabolic pathways associated with fasting and regulation of AA balance.

### 3.3 LAT2KO Preserves Muscular Mass During Fasting and Decreases Proteasomal Activity and Autophagy

**FIGURE 3:**
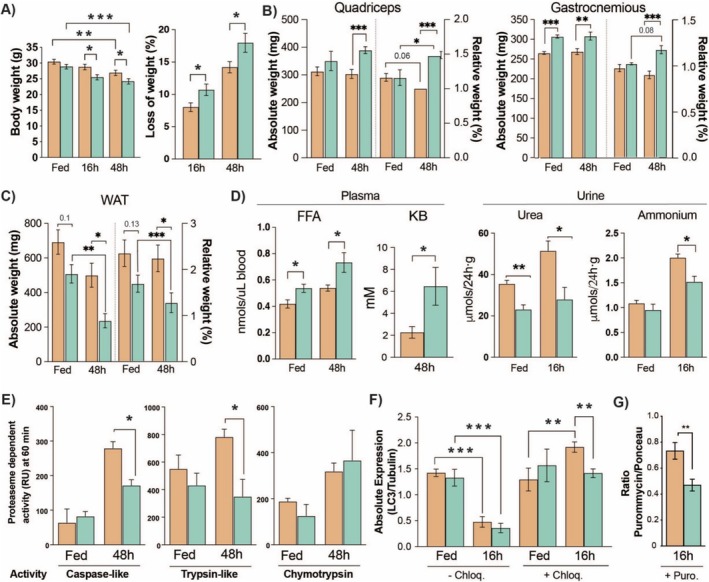
Characterisation of LAT2 knockout mouse body composition and muscle phenotype during fasting. (A) Body weight and percentage body weight loss after 16‐ and 48‐h fasting (*N* = 16 per genotype). (B) Absolute and relative tissue weight of gastrocnemius, quadriceps, and (C) abdominal white adipose tissue (WAT) (*N* = 8–10 per genotype and condition). (D) Free fatty acids (FFAs) and ketone bodies (KBs) in plasma samples, and urine urea and ammonium content at 24 h (*N* = 8 per genotype and condition). (E) Muscle proteasome activity data measured via fluorometric assay of fed and 48‐h‐fasted muscles (*N* = 8 per genotype and condition) for Caspase‐like, Trypsin‐like and Chymotrypsin activity. (F) Western blot quantification of LC3 expression, normalised to tubulin, in gastrocnemius muscles of fed and 16‐h‐fasted mice preinjected with saline (−Chloq) or chloroquine (+Chloq) (*N* = 5 per genotype and condition). (G) Quantification of protein synthesis by SUnSET of 16‐h‐fasted mice (*N* = 7 per genotype). (A–G) Mean + SEM of WT (orange) and LAT2 knockout (KO) (turquoise) samples are presented. Wilcoxon rank sum test *p* values represented by *< 0.05, **< 0.01 and ***< 0.001.

Fasting induced significant phenotypic differences in WT and LAT2KO mice (Figure 3). Significant weight loss, with an 18% reduction in total body weight, was seen in the LAT2KO group, compared with a 14% loss in 48‐fasted WT mice (Figure 3A and Figure S2). Prior to fasting, LAT2KO mice showed a trend to have greater muscle mass and lower body fat than WT mice, a pattern that was reinforced following fasting (Figure 3B,C). The extensive white adipose tissue (WAT) loss and comparative maintenance of muscle mass after long‐term fasting observed in the LAT2KO pointed to altered catabolism where lipolysis is increased and proteolysis reduced. The increased concentrations of plasma KBs and FFAs, markers of lipolysis, in fasted LAT2KO animals compared with fasted WT mice aligned with reduced WAT (Figure 3D). The urea and ammonium content of urine, markers of proteolysis, were also significantly lower in the fasted LAT2KO mice (Figure 3D). Subsequently, we measured ubiquitin‐linked proteasome and autophagy activity following a 48‐h fast and observed significantly lower levels of caspase‐like and trypsin‐like proteasome activity in LAT2KO compared to those of WT mice following fasting (Figure 3E). Furthermore, fasted LAT2KO mice displayed a 0.7‐fold decrease in LC3 in the presence of chloroquine, indicating they harboured lower autophagic flux levels than WT animals (Figure 3F). Unexpectedly, muscle protein synthesis during fasting was reduced 40% in LAT2KO mice compared with fasted WT (Figure 3G).

To further investigate the effects of LAT2 deficiency on muscle phenotype, both muscle morphology and tone were analysed. Histological analysis of gastrocnemius muscle fibres did not show any gross differences in the patterns of staining (Figure S3A), but the cross‐sectional area (CSA) of LAT2KO gastrocnemius muscle exhibited a greater distribution of larger sized fibres and a higher median size than WT (Figure S3B). Additionally, we performed a grip‐strength test and assessed the animal’s overall fitness and time to fatigue during a boost‐speed treadmill workout. LAT2KO mice did not show any impairment in muscular strength (Figure S3C) but displayed a significant reduction in performance during the treadmill workout compared to that of the WT (Figure S3D). As mitochondria are the main source of cellular energy and adapt to meet physiological demands, we analysed both muscle mitochondrial function and content. An analysis of mitochondrial DNA content did not show significant differences between LAT2KO and WT cells (Figure S3E), and an assessment of mitochondrial morphology by electron microscopy did not reveal any substantial structural differences (data not shown). In comparison, high‐resolution respirometry performed with fresh muscle fibres revealed significant differences in their mitochondrial respiration rates. Although no differences were observed in the leak state (malate + pyruvate), significantly lower Complex I activation (ADP + glutamate), Complex II activation (succinate) and maximal respiration induction rates were found in LAT2KO compared with WT muscle (Figure S3F).

### 3.4 Glutamine Accumulation Inhibits Fasting‐Induced Proteolysis in Skeletal Muscle

**FIGURE 4:**
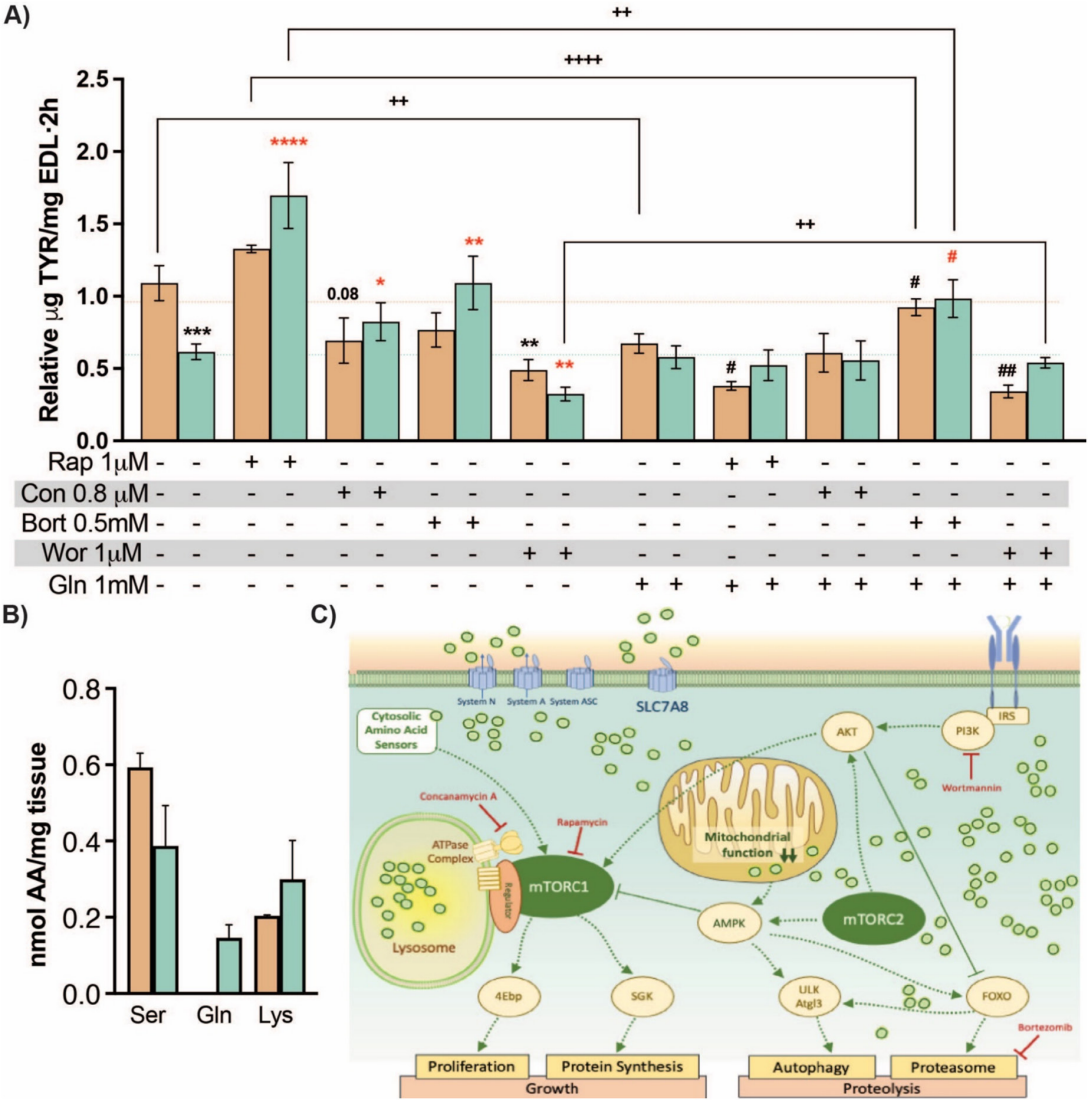
Proteolysis activity measured ex vivo in EDL muscles. (A) Tyrosine release quantification of overnight‐fasted EDL muscles incubated for 2 h in the presence or absence of rapamycin (Rap), concanamycin A (Con), bortezomib (Bort), wortmannin (War), and/or glutamine (Gln). All incubation protein synthesis was inhibited with 25 mM cycloheximide. Mean +/− SEM of 6–12 WT (orange) or LAT2KO (turquoise) measurements are presented. Wilcoxon rank sum test *p* values are presented: * versus AA‐free medium; # versus + Gln, where black symbols indicate comparison versus the WT and in red versus the LAT2KO of each condition; #;*< 0.05, ##,**,++ < 0.01, *** < 0.001, and ****, ++++ < 0.0001. (B) AA content of EDL incubated in AA‐free medium per milligramme of tissue (*N* = 6 per genotype). (C) Diagram of described signalling systems involving AAs and mTORC1 pathway during catabolic state. Positive regulation is indicated by green dashed arrows, negative regulation by green lines, and inhibitors used for the ex vivo experiment are in red. Circles represent AAs.

To directly quantify the effects of LAT2 deficiency on muscle proteostasis, we assessed the net proteolysis flux ex vivo by measuring tyrosine release from EDL muscle after overnight fasting (16 h). The results showed that proteolysis levels were reduced to nearly 50% in LAT2KO mice in comparison with WT animals (Figure 4A). Subsequently, when the AA content of EDL muscles was measured, only LAT2KO muscles incubated in AA‐free media showed a substantial difference in Gln content, which was determined to be 0.2 nmol/g tissue, whereas Gln was undetectable in WT muscle (Figure 4B). To determine if intramuscular Gln accumulation drove the inhibition of proteolysis, WT and LAT2KO EDL muscles were incubated in the presence of 1mMGln. Gln supplementation reduced proteolysis in WT muscle to a level comparable to that seen in the LAT2KO muscle (Figure 4A). To further characterise the Gln‐induced inhibition of proteolysis, fasted EDL muscles from both genotypes were incubated ex vivo in the presence of several inhibitors: rapamycin, an mTORC1 inhibitor; concanamycin A, a lysosome/vATPase inhibitor; bortezomib, a proteasome inhibitor; and wortmannin, a PI3K inhibitor. The addition of rapamycin to LAT2KO muscle rescued the phenotype and led to enhanced rates of proteolysis in both WT and LAT2KO muscle fibres, indicating that the inhibition of proteolysis in LAT2KO muscles was mTORC1‐dependent. Concanamycin A and bortezomib were used to inhibit autophagy (by disrupting lysosomal membranes) and UPS, respectively. WT muscles incubated with either inhibitor showed a slight decrease in proteolysis, whereas LAT2KO muscles showed a significant increase in proteolysis in the presence of both inhibitors, including a 2‐fold increase in the presence of bortezomib. Additionally, PI3K/AKT signalling was inhibited by wortmannin, and an additional inhibitory effect was observed in both genotypes that halved proteolysis activity (Figure 4A).

Coincubation of muscles with Gln and rapamycin did not rescue the phenotype of the WT or LAT2KO muscles, further confirming that Gln supplementation inhibited proteolysis via an mTORC1‐independent pathway (Figure 4A). When autophagy was inhibited in the presence of concanamycin A and external Gln, there were no observable changes, regardless of the genotype. In contrast, a significant increase in proteolysis was observed for both genotypes when muscles were coincubated with bortezomib and Gln, with the same effect observed as when LAT2KO muscles were incubated with bortezomib alone. Notably, wortmannin together with Gln increased proteolysis inhibition by Gln in WT muscle but not in the corresponding LAT2KO muscle (Figure 4A).

Overall, we provide evidence that the accumulation of intramuscular Gln due to the absence of the LAT2 transporter inhibits the two main proteolysis systems, autophagy and UPS, through the inhibition of mTORC1 signalling. Additionally, external Gln supplementation also inhibited proteolysis through an alternate mTORC1‐independent pathway (Figure 4A).

### 3.5 Increased mTORC1 Recruitment to the Lysosome in Fasted LAT2KO Muscles

**FIGURE 5:**
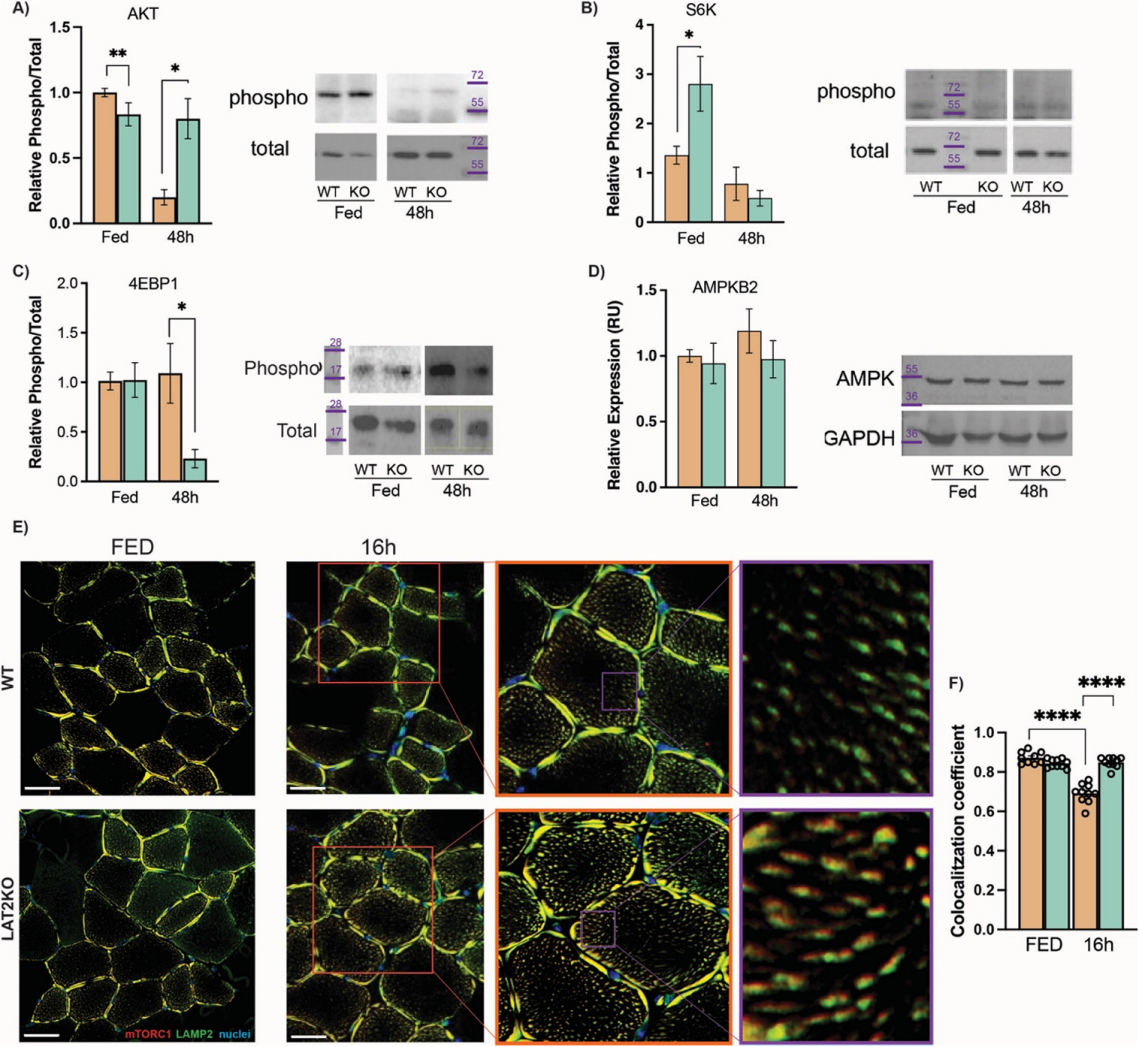
mTORC1 signalling and recruitment to the lysosome in fasted LAT2 knockout mouse muscles (A‐D) Quantification of western blot of gastrocnemius muscle from fed and 48‐h fasted mice. Mean +/− SEM of *n* = 6 samples. (A) Phospho‐ and total AKT, (B) phospho‐ and total S6K, (C) phospho‐ and total 4EBp, and (D) total AMPK expression normalised to GAPDH. (E) Confocal images of immunofluorescence staining of gastrocnemius muscle of Fed and 16‐h fasted WT and LAT2KO samples using Lamp1 (green) to stain lysosome, mTORC1 (red), and DAPI nuclear staining (blue). Scale bar in white on the bottom left represents 20 mm. (F) Colocalization of mTOR and Lamp1 quantification determined by Pearson’s coefficient. (A, B, C, and F) Mean + SEM of WT (orange) and LAT2KO (turquoise) samples are presented. Wilcoxon rank sum test *p* values are represented by *< 0.05, **< 0.01 and ****< 0.0001.

As mTORC1 is the central nutrient‐dependent regulator of cellular metabolism, we next examined the upstream and downstream signalling pathways involved in mTORC1 regulation in fed and 48‐h fasted muscle. Compared to WT, LAT2KO muscle exhibited increased Akt phosphorylation and decreased 4EBP1 protein phosphorylation after fasting (Figure 5A–C). In contrast, AMPK expression and S6K phosphorylation remained unaltered in fasted LAT2KO muscles (Figure 5D).

Given the potential activation of mTORC1 through the lysosomal‐Rag/GTPase complex by AAs [6], the localization of mTORC1 at lysosomes in fed and fasted muscles was analysed. Gastrocnemius muscle sections were stained with a nuclear marker, and mTORC1 and Lamp1 antibody labelling was analysed by confocal microscopy (Figure 5E). No significant differences were observed in mTORC1–Lysosome colocalization between WT and LAT2KO in the fed state (Figure 5F). In contrast, following a 16‐h fast, WT muscles showed a significant decrease in the amount of mTORC1 localised at the lysosome, whereas fasted LAT2KO muscle maintained its levels of mTORC1‐lysosome complex formation, which were similar to those observed in the fed state (Figure 5F).

### 3.6 LAT2KO Phenotype in Chronic Diseases Associated With Increased Proteolysis

Following the observation of sustained inhibition of proteolysis in the LAT2KO mice, we explored whether this effect could be therapeutically advantageous in diseases with impaired protein homeostasis, including Lewis’ CA, STZ‐induced T1D and ageing‐induced sarcopenia. Initial observations of mouse models for these diseases revealed that *Slc7a8* expression in WT muscle was increased 2‐fold in the T1D model and aged groups, whereas expression was unaltered in the CA model, compared with young control mice (Figure S4A).

The CA model did not show genotype‐dependent differences in the severity of the disease, as measured by tumour size or muscle mass (Figure S4B). T1D was successfully induced by STZ injection and resulted in a remarkable increase in blood glucose in both genotypes (Figure S4C). However, no differences in the loss of muscle mass or mass from other organ systems, including the liver and WAT, were observed (Figure S4D). Subsequent measurement of muscle proteasome activity in both CA and T1D models was conducted, but no significant differences were observed between LAT2KO and WT animals (Figure S4E).

**FIGURE 6:**
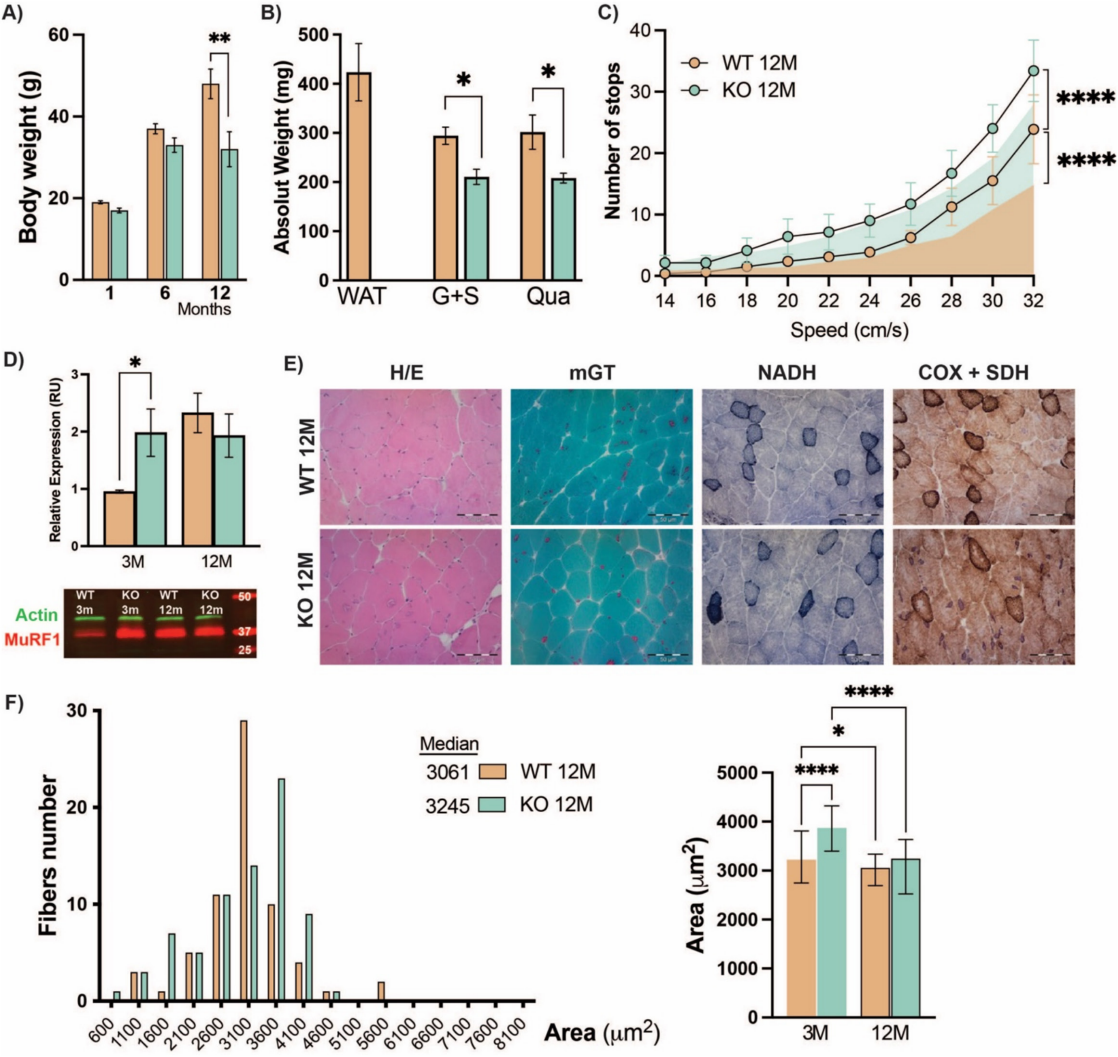
Age‐related phenotype characterisation in LAT2 knockout mouse muscles. (A) Body weight of 1‐, 6‐, and 12‐month‐old WT and LAT2KO mice (*N* = 12 mice per genotype). (B) Absolute tissue weight of abdominal WAT, G + S muscle, and Qua muscle samples from 12‐month‐old WT and LAT2KO mice (*N* = 6 samples per genotype). (C) Results of treadmill increasing speed test performed for a fixed distance and after training session for 12‐month‐old (12 M) WT and LAT2KO animals; as reference, the area under the curve (AUC) of young WT and LAT2KO (3 M) is represented in turquoise and orange respectably (*N* = 12 mice per genotype). Number of stops represent each time the mice fell from the running belt into the shock chamber. (D) Quantification and western blot of Murf1 (*N* = 4 per genotype and age) of gastrocnemius muscle protein extract. (E) Histopathology of 6 WT and 6 age‐matched LAT2KO gastrocnemius muscle at 12 months of age. Haematoxylin and eosin (H/E), modified Gömori trichrome (mGT), reduced nicotinamide adenine dinucleotide dehydrogenase‐tetrazolium reductase (NADH), and cytochrome c oxidase + succinic dehydrogenase (COX + SDH) was used to evaluate muscle tissue. Scale bar for all staining represent 50 mm. (F) Cross‐sectional area (CSA) distribution and mean of gastrocnemius muscle from 12‐month‐old WT and LAT2KO mice (*N* = 8 per genotype). (A–F) Mean + SEM of WT (orange) and LAT2KO (turquoise) samples are presented. Wilcoxon rank sum test *p* values are represented by *< 0.05, **< 0.01, and ****< 0.0001.

LAT2 mutations have been described as contributing to the early onset of both hearing loss and cataract growth in human populations [18, 26]. Here, we explored whether LAT2 deficiency results in an early onset of age‐related muscle phenotype. LAT2KO animals showed reduced body weight gain during their lifespan, mainly attributable to their reduced adiposity (WAT tissue was not present in aged‐LAT2KO mice) compared to age‐matched WT animals (Figure 6A,B). Muscle performance during the boost‐speed test showed differences between the young and 12 M old WT; however, young LAT2KO mice performed poorly in the test and had characteristics similar to those seen in the ageing groups (Figure 6C). Notably, the expression of Murf1, a marker found in aged muscle, was increased in young LAT2KO and was present at levels comparable to those observed in both genotypes in the ageing animals (Figure 6D). Despite these findings, neither histological analysis nor CSA of aged muscles revealed any significant differences between the genotypes (Figure 6E,F).

## 4 Discussion

Through a series of experiments combining the LAT2KO model and dietary intervention, we demonstrated that the LAT2 transporter is critical for the regulation of Gln content in skeletal muscles. LAT2 deficiency led to the intracellular accumulation of Gln and inhibited muscle proteolysis during fasting. Importantly, our study elucidated how Gln‐dependent proteolysis inhibition functions through the reduction of both proteasomal and autophagic activities in an mTORC1‐dependent manner. We also observed that LAT2 transporter deficiency resulted in the manifestation of a premature ageing‐related phenotype in mouse muscle tissues.

The capacity of AA transporters to promote AA accumulation depends on the presence of cotransported ions, the electrochemical gradient, and the cellular potential. Systems N and y^+^L concentrate Gln up to 5‐fold and System A up to 12‐fold [27]. In contrast, System L and System ASC function as obligate exchangers with a 1:1 stoichiometry. We observed a 3‐ to 6‐fold increase in intramuscular Gln in the absence of LAT2, indicating that other transporters with a 1:1 stoichiometry present in the muscle, such as Systems ASC or L, cannot compensate for the loss of LAT2 and Gln efflux (Figure 1D and Table S4). The increased basic AA content (Lys and Arg) was intervention intended (Figure 1D and Table S4). Neutral intramuscular AA imbalance might affect tertiary transport mediated by the y + L system, which mediates electroneutral exchange of dibasic AA in exchange for neutral amino in a Na^+^‐dependent manner [28].

After 48 h of fasting, LAT2KO animals retained a greater amount of muscle mass but exhibited substantial reductions in body fat compared with WT animals (Figure 3A,B). These changes were associated with increased lipolysis, as LAT2KO animals exhibited elevated levels of FFA and KB in the bloodstream (Figure 3C). Muscle proteolysis leads to an increase in nitrogen waste products such as urea [29]. Concordant with the muscle preservation observed in fasted LAT2KO mice, the urea and ammonium contents of urine were increased by fasting in the WT group but were significantly reduced in the fasted LAT2KO group (Figure 3C). The Gln released from skeletal muscle in response to starvation is critical for transorgan metabolic flux, including ureagenesis in the liver and as a precursor for gluconeogenesis in both the kidneys and liver [30]. However, the livers of LAT2KO mice and blood Gln content remained unchanged in Fed and fasted states (Tables S2 and S3). Cit levels, which enhance the urea cycle, were significantly higher in LAT2KO muscles, suggesting that the reduced nitrogen clearance was due to reduced proteolysis rather than an impairment of the urea cycle (Figure 1D).

In this study, we found a significant reduction in fasting‐induced muscle proteolysis in LAT2KO mice, with evidence of reduced activity in both the ubiquitin–proteasome and autophagy systems (Figure 3E,F). Moreover, protein synthesis was also reduced by 40% in fasted LAT2KO muscles (Figure 3G). Phosphorylation of 4E‐BP was decreased, and no differences were observed in S6K levels, which remained similar to the fasted WT mice (Figure 5A–D). Our ex vivo measurements of proteolysis in the presence/absence of several inhibitors and Gln outlined the complex regulatory crosstalk occurring between the two major proteolytic pathways (UPS and autophagy) in a context where protein synthesis was inhibited and AA content controlled. In these settings, we demonstrated that inhibition of proteolysis in LAT2KO mice is regulated via a mTORC1‐dependent mechanism, whereas external Gln supplementation inhibited proteolysis by an alternate signalling mechanism (Figure 4A). Gln has been described to regulate mTORC1‐dependent autophagy during AA deprivation in C2C12 mouse myotubes [31]. Additionally, as described in [32], increases in the intracellular AA content promote mTORC1 migration to the lysosome, driving its activation. Immunofluorescence staining revealed increased mTORC1 localization at the lysosome in fasted LAT2KO muscles, coincident with increased levels of intracellular Gln (Figure 5E). The mechanisms by which AAs regulate mTOR‐lysosome interactions and metabolic signalling remain poorly understood. A recent preprint by Kolaczkowski et al. [33] characterised lysosome positioning and mTOR activation by various AAs in a cell model. They observed that some AAs can induce mTOR activation and others influence lysosome positioning. In this work, we conclude that LAT2KO intramuscular Gln accumulation inhibits fasting‐mediated proteolysis by steady mTORC1 activation through its binding to the lysosome. Moreover, we could not exclude a synergistic effect of other AAs accumulated in the LAT2KO muscle such as Ser, Lys, or Arg (Figure 1D) explaining the decrease observed in both protein degradation and synthesis simultaneously.

LAT2 expression in skeletal muscle was found to be higher in fast‐twitch (white) fibres than slow‐twitch (red) fibres (Figure 1A). The muscles analysed in this study (EDL, gastrocnemius, and quadriceps) are composed of > 80% white fibres, which are characterised by their low mitochondrial oxidative respiratory (OXPHOS) capacity, primarily relying on glycolysis for ATP production [34]. Despite having an equivalent load of mitochondrial DNA per cell, LAT2KO cells exhibited a 20% decrease in OXPHOS compared to WT (Figure S3F). Limited amounts of Gln‐derived metabolites, such as Cit, ornithine and proline, have been observed in cells with altered mitochondrial function, negatively impacting cellular redox and ammonia detoxification. However, in the LAT2KO mice, these metabolites were present in excess (Table S1). Mitochondrial functional validation was performed using permeabilized fibres and substrate titration, allowing us to exclude metabolite‐related limitations as the cause for the observed mitochondrial dysfunction. Alternatively, a reduction in proteolysis in the LAT2KO may have inhibited the clearance of damaged mitochondria by autophagy or mitophagy [35].

To understand if the LAT2KO cells accumulated defective or dysfunctional mitochondria, we analysed their mitochondrial morphology by electron microscopy. Ultrastructural analysis of WT and LAT2KO animals did not show any relevant abnormalities, except for the presence of tubular aggregates in both genotypes. No mitochondrial abnormalities in the number or morphology of mitochondria were seen in the LAT2KO muscles. It is well known that cells modulate their mitochondrial function in response to excess levels of intracellular AAs, but the mechanism by which cells respond to toxic levels of AAs remains unclear [36, 37]. However, the recently discovered mitochondrial‐derived compartments, secondary structures that selectively sequester mitochondrial transporters in adjacent vesicles and limit transporter function, are one potential mechanism [38].

Persistent mTORC1 activation and decreased autophagy have been linked to age‐related and progressive diseases [39, 40, 41]. Age‐related phenotypes, such as the reduced muscle tone and reduced body weight gain associated with decreased adiposity, were observed in the LAT2KO mice (Figure 6). Together with our previous finding that LAT2 mutations cause the early onset of ageing‐related diseases, including hearing loss and cataracts [18, 26], our evidence suggests that understanding the contribution of Gln accumulation and proteolysis regulation to these age‐related diseases may be of significant clinical interest.

In conclusion, our data demonstrate that LAT2 plays a pivotal role in controlling Gln efflux in skeletal muscle. The absence of this transporter results in high intracellular Gln levels and impaired proteolysis by decreasing the proteasome and autophagy regulated by mTORC1 recruitment to the lysosome. Understanding the role of LAT2 in different cell types and metabolic conditions may have significant clinical implications for age‐related diseases and metabolic disorders. Recent work by [17] described the key role of LAT2 in the activation of ILC2s, as mice lacking this transporter had significantly reduced numbers of ILC2s and showed attenuated responses to allergens as a result of compromised mitochondrial OXPHOS and mTORC1 signalling. Additionally, a diet‐induced obesity model in LAT2KO mice showed significantly reduced lipid accumulation [42]. Together, these findings imply that further research on LAT2 is warranted, as it has been largely overshadowed by its better‐known and better‐characterised cousin, the Slc7a5 (LAT1) L‐type alternate transporter. Together, our results provide new and interesting insights into the pivotal role of the LAT2 transporter in governing Gln content in skeletal muscle and its impact on multiple physiological processes.

## 5 Study Limitations

Under conditions of nutrient deprivation, the LAT2KO group metabolically adapted to compensate for reduced proteolysis by accelerated lipolysis in WAT. It remains uncertain if this accelerated lipolysis was a cause or consequence of decreased autophagic and proteasomal activity in the muscle, and a tissue‐specific model would be needed to address this issue.

## Ethics Statement

All protocols used in this study were reviewed and approved by the Institutional Animal Care and Use Committee at IDIBELL and carried out in a facility accredited by the Association for the Assessment and Accreditation of Laboratory Animal Care International (AAELAC). Mice procedures were preplanned and executed according to the highest scientific, humane and ethical principles.

## Conflicts of Interest

The authors declare no conflicts of interest.

## Supporting information


**Figure S1.** RNA‐Seq data analysis. (A) Venn diagram representing DEG’s for each comparison. (B) Heat map demonstrating clustering of top 2000 most variable up‐ and down‐regulated genes for individual animals from WT and LAT2KO in fed and 48‐h fasted groups. (C) PCA plot indicating principal components that efficiently segregate fasting (PC1) and genotype (PC4).
**Figure S2.** Absolute tissue weight of fed and 16‐ and 48‐h fasted mice. Mean + SEM of absolute tissue weight of liver, kidneys, pancreas, spleen, and brain from 6 to 8 mice. Mean + SEM of relative tissue weight to mice total weight of gastrocnemius, quadriceps, and abdominal adipose tissue (WAT) from 6 to 8 mice. Wild type (orange) and LAT2 knockout (turquoise) samples are presented. Wilcoxon rank sum test *p* values are represented by *< 0.05 and **< 0.01.
**Figure S3.** Effects of LAT2 knockout on muscle morphology and function. (A) Histopathology of WT and LAT2KO gastrocnemius muscle sections. Haematoxylin and eosin (H/E), modified Gömori trichrome (mGT), Periodic acid Schiff technique (PAS), reduced nicotinamide adenine dinucleotide dehydrogenase‐tetrazolium reductase (NADH), cytochrome c oxidase (COX), and succinic dehydrogenase (SDH) were used to evaluate muscle tissue from fed animals (*N* = 8 per genotype). Scale bar represents 50 mm for H/E, PAS, NADH and SDH; 20 mm for mGT and 100 mm for COX staining. (B) Cross sectional area (CSA) distribution and means (dots represent each quantified area) of fed gastrocnemius muscle (*N* = 6 per genotype). (C) Grip strength test results showing minimum (min) and maximum (max) strength recorded per animal and assessed in triplicate. (D) Results of treadmill increasing speed test performed for a fixed distance and after training session (*N* = 12 mice per genotype). Number of stops represent each time the mice felt from the running belt into the shock chamber. (E) Mitochondrial copy numbers measured by real time PCR. Median and percentiles are presented (*N* = 12 per genotype). (F) Oxygen composition analysis by high‐resolution respirometry of permeabilized muscle according to tissue weight. GM: glutamate 3+ pyruvate (basal), GMD: addition of ATP (CI), GMDS: addition of succinate (CII), FCCP: Addition of uncouplers (CI + CII max), and Rot: addition of rotenone inhibitor (CII max). (B–F) Mean + SEM of WT (orange) and LAT2 knockout (KO) (turquoise) samples are presented. Wilcoxon rank sum test *p* values are represented by *< 0.05, ***< 0.001 and ****< 0.0001.
**Figure S4.** Effect of LAT2 knockout on chronic diseases with increased proteolysis. (A) Analysis of *Lat2* expression by real time PCR in muscle from aged, Type 1 diabetes (T1D), Lewis’s cancer‐cachexia (CA), and young wild type (WT) mice normalised to GAPDH. (B) Relative tissue weight normalised to initial body weight (IBW) of CA model for gastrocnemius + soleus (G + S), tibialis (Tib), quadriceps (Qua), abdominal white adipose tissue (WAT), and tumour of 6 mice per genotype injected with CA cell suspension. (C) Blood glucose levels in Fed and streptozotocin‐induced T1D mice. (D) Relative tissue weight normalised to body weight of T1D model for Qua, gastrocnemius (Gas), soleus (Sol), spleen (Spl), Liver (Liv), and WAT of 8 T1D mice per genotype. **(E)** Gastrocnemius muscle proteasome activity measurements by fluorometric assay of Fed, CA, and T1D models (*N* = 6 per genotype and condition). (B–E) WT (orange) and LAT2 knockout (KO) (turquoise) samples are presented. Wilcoxon rank sum test p‐values are represented by *< 0.05 and **< 0.01.
**Table S1.** AA content of skeletal muscle AA content of G + S muscles in fed and 16‐ and 48‐h fasted mice, per mg of tissue (*N* = 6–12 per condition and genotype); mean + SEM and ratios (fold change, LAT2KO vs. WT) are represented. Wilcoxon rank sum test *p*‐values are represented by *< 0.05, **< 0.01, and ***< 0.001.
**Table S2.** AA content of liver AA content of liver in fed and 16‐h fasted mice, per milligrammes of tissue (*N* = 6–12 per condition and genotype); mean + SEM and ratios (fold change, LAT2KO vs. WT) are presented. Wilcoxon rank sum test *p* values are represented by *< 0.05 and **< 0.01.
**Table S3.** Amino acid content of blood plasma amino acid (AA) content in Fed and 16‐ and 48‐h fasted mice, according to volume of plasma (*N* = 6–12 per condition and genotype); mean + SEM and ratios (fold change, LAT2KO vs. WT) are presented. Wilcoxon rank sum test *p* values are represented by *< 0.05 and ***< 0.001.
**Table S4.** Gene expression of amino acid transporters in G + S muscle Gene expression of Fed and 16‐h fasted (FAST) WT and LAT2KO mice, analysed using Roche UPL‐System (*N* = 6 per condition and genotype). Substrate: AAs are shown as single‐letter codes, AA0: neutral amino acids. Mean + SEM of fold change (LAT2KO vs. WT) is presented. Wilcoxon rank sum test *p* values are represented by *< 0.05, **< 0.01, and ***< 0.001.
**Table S5.** Methodology details. (A) Primers used for real‐time PCR. Symbol of the gene name. Ref. for mRNA gene sequence. Primer’s sequence shown as 5′ to 3′. Roche UPL probe identifier and amplicon size. (B) Antibodies used for western blot and immunofluorescence. Protein name. Reference for commercial details (catalogue number, company). WB dil indicates dilutions and buffer used for western blot. IF dil indicates dilution used for immunofluorescence and (if) secondary antibody (Ab) used and its dilution (Dil sec).

## References

[1] C. Hambly and J. R. Speakman , “Contribution of Different Mechanisms to Compensation for Energy Restriction in the Mouse,” Obesity Research 13, no. 9 (2005): 1548–1557. 16222057 10.1038/oby.2005.190

[2] M. Sandri , “Protein Breakdown in Muscle Wasting: Role of Autophagy‐Lysosome and Ubiquitin‐Proteasome,” International Journal of Biochemistry & Cell Biology 45, no. 10 (2013): 2121–2129. 23665154 10.1016/j.biocel.2013.04.023 PMC3775123

[3] J. H. Lee , S. Park , E. Kim , and M. J. Lee , “Negative‐Feedback Coordination Between Proteasomal Activity and Autophagic Flux,” Autophagy 15, no. 4 (2019): 726–728. 30689498 10.1080/15548627.2019.1569917 PMC6526830

[4] S. Cohen , J. A. Nathan , and A. L. Goldberg , “Muscle Wasting in Disease: Molecular Mechanisms and Promising Therapies,” Nature Reviews. Drug Discovery 14, no. 1 (2015): 58–74. 25549588 10.1038/nrd4467

[5] M. Shimobayashi and M. N. Hall , “Multiple Amino Acid Sensing Inputs to mTORC1,” Cell Research 26, no. 1 (2016): 7–20. 26658722 10.1038/cr.2015.146 PMC4816134

[6] G. Y. Liu and D. M. Sabatini , “mTOR at the nexus of Nutrition, Growth, Ageing and Disease,” Nature Reviews Molecular Cell Biology 21, no. 4 (2020): 183–203. 31937935 10.1038/s41580-019-0199-y PMC7102936

[7] R. L. Wolfson , L. Chantranupong , R. A. Saxton , et al., “Sestrin2 Is a Leucine Sensor for the mTORC1 Pathway,” Science 351, no. 6268 (2016): 43–48. 26449471 10.1126/science.aab2674 PMC4698017

[8] A. Parmigiani , A. Nourbakhsh , B. Ding , et al., “Sestrins Inhibit mTORC1 Kinase Activation Through the GATOR Complex,” Cell Reports 9, no. 4 (2014): 1281–1291. 25457612 10.1016/j.celrep.2014.10.019 PMC4303546

[9] D. M. Gwinn , D. B. Shackelford , D. F. Egan , et al., “AMPK Phosphorylation of Raptor Mediates a Metabolic Checkpoint,” Molecular Cell 30, no. 2 (2008): 214–226. 18439900 10.1016/j.molcel.2008.03.003 PMC2674027

[10] D. F. Egan , D. B. Shackelford , M. M. Mihaylova , et al., “Phosphorylation of ULK1 (hATG1) by AMP‐Activated Protein Kinase Connects Energy Sensing to Mitophagy,” Science 331, no. 6016 (2011): 456–461. 21205641 10.1126/science.1196371 PMC3030664

[11] D. L. Oxender and H. N. Christensen , “Evidence for Two Types of Mediation of Neutral and Amino‐Acid Transport in Ehrlich Cells,” Nature 197 (1963): 765–767. 13940861 10.1038/197765a0

[12] M. Pineda , E. Fernández , D. Torrents , et al., “Identification of a Membrane Protein, LAT‐2, That Co‐Expresses With 4F2 Heavy Chain, an L‐Type Amino Acid Transport Activity With Broad Specificity for Small and Large Zwitterionic Amino Acids,” Journal of Biological Chemistry 274, no. 28 (1999): 19738–19744. 10391915 10.1074/jbc.274.28.19738

[13] M. J. Rennie , A. Ahmed , S. E. O. Khogali , S. Y. Low , H. S. Hundal , and P. M. Taylor , “Glutamine Metabolism and Transport in Skeletal Muscle and Heart and Their Clinical Relevance,” Journal of Nutrition 126, no. 4 Suppl (1996): 1142S–1149S. 8642447 10.1093/jn/126.suppl_4.1142S

[14] P. Nicklin , P. Bergman , B. Zhang , et al., “Bidirectional Transport of Amino Acids Regulates mTOR and Autophagy,” Cell 136, no. 3 (2009): 521–534. 19203585 10.1016/j.cell.2008.11.044 PMC3733119

[15] Z. Wang , B. Li , S. Li , et al., “Metabolic Control of CD47 Expression Through LAT2‐Mediated Amino Acid Uptake Promotes Tumor Immune Evasion,” Nature Communications 13, no. 1 (2022): 6308. 10.1038/s41467-022-34064-4 PMC9588779 36274066

[16] M. Feng , G. Xiong , Z. Cao , et al., “LAT2 Regulates Glutamine‐Dependent mTOR Activation to Promote Glycolysis and Chemoresistance in Pancreatic cancer,” Journal of Experimental & Clinical Cancer Research 37, no. 1 (2018): 274. 30419950 10.1186/s13046-018-0947-4 PMC6233565

[17] S. K. Panda , D. H. Kim , P. Desai , et al., “SLC7A8 Is a key Amino Acids Supplier for the Metabolic Programs That Sustain Homeostasis and Activation of Type 2 Innate Lymphoid Cells,” Proceedings of the National Academy of Sciences of the United States of America 119, no. 46 (2022): e2215528119. 36343258 10.1073/pnas.2215528119 PMC9674248

[18] M. Espino Guarch , M. Font‐Llitjós , S. Murillo‐Cuesta , et al., “Mutations in L‐Type Amino Acid Transporter‐2 Support SLC7A8 as a Novel Gene Involved in Age‐Related Hearing Loss,” eLife 7 (2018): e31511. 29355479 10.7554/eLife.31511 PMC5811215

[19] M. Mauthe , I. Orhon , C. Rocchi , et al., “Chloroquine Inhibits Autophagic Flux by Decreasing Autophagosome‐Lysosome Fusion,” Autophagy 14, no. 8 (2018): 1435–1455. 29940786 10.1080/15548627.2018.1474314 PMC6103682

[20] E. K. Schmidt , G. Clavarino , M. Ceppi , and P. Pierre , “SUnSET, a Nonradioactive Method to Monitor Protein Synthesis,” Nature Methods 6, no. 4 (2009): 275–277. 19305406 10.1038/nmeth.1314

[21] S. Busquets , M. Toledo , M. Orpí , et al., “Myostatin Blockage Using actRIIB Antagonism in Mice Bearing the Lewis Lung Carcinoma Results in the Improvement of Muscle Wasting and Physical Performance,” Journal of Cachexia, Sarcopenia and Muscle 3, no. 1 (2012): 37–43. 22450815 10.1007/s13539-011-0049-z PMC3302990

[22] C. Carducci , M. Birarelli , V. Leuzzi , G. Santagata , P. Serafini , and I. Antonozzi , “Automated Method for the Measurement of Amino Acids in Urine by High‐Performance Liquid Chromatography,” Journal of Chromatography A 729, no. 1–2 (1996): 173–180. 9004938 10.1016/0021-9673(95)00964-7

[23] X. Lornage , V. Schartner , I. Balbueno , et al., “Clinical, Histological, and Genetic Characterization of PYROXD1‐Related Myopathy,” Acta Neuropathologica Communications 7, no. 1 (2019): 138. 31455395 10.1186/s40478-019-0781-8 PMC6710884

[24] S. X. Ge , E. W. Son , and R. Yao , “iDEP: An Integrated Web Application for Differential Expression and Pathway Analysis of RNA‐Seq Data,” BMC Bioinformatics 19, no. 1 (2018): 534. 30567491 10.1186/s12859-018-2486-6 PMC6299935

[25] S. Broer , “Amino Acid Transport Across Mammalian Intestinal and Renal Epithelia,” Physiological Reviews 88, no. 1 (2008): 249–286. 18195088 10.1152/physrev.00018.2006

[26] E. B. Knöpfel , C. Vilches , S. M. Camargo , et al., “Dysfunctional LAT2 Amino Acid Transporter Is Associated With Cataract in Mouse and Humans,” Frontiers in Physiology 10 (2019): 688. 31231240 10.3389/fphys.2019.00688 PMC6558864

[27] G. Gauthier‐Coles , J. Vennitti , Z. Zhang , et al., “Quantitative Modelling of Amino Acid Transport and Homeostasis in Mammalian Cells,” Nature Communications 12, no. 1 (2021): 5282. 10.1038/s41467-021-25563-x PMC8421413 34489418

[28] H. S. Hundal and P. M. Taylor , “Amino Acid Transceptors: Gate Keepers of Nutrient Exchange and Regulators of Nutrient Signaling,” American Journal of Physiology. Endocrinology and Metabolism 296, no. 4 (2009): E603–E613. 19158318 10.1152/ajpendo.91002.2008 PMC2670634

[29] V. Walker , “Ammonia Metabolism and Hyperammonemic Disorders,” Advances in Clinical Chemistry 67 (2014): 73–150. 25735860 10.1016/bs.acc.2014.09.002

[30] Y. He , T. B. M. Hakvoort , S. E. Köhler , et al., “Glutamine Synthetase in Muscle Is Required for Glutamine Production During Fasting and Extrahepatic ammonia Detoxification,” Journal of Biological Chemistry 285, no. 13 (2010): 9516–9524. 20064933 10.1074/jbc.M109.092429 PMC2843202

[31] H. W. S. Tan , A. Y. L. Sim , and Y. C. Long , “Glutamine Metabolism Regulates Autophagy‐Dependent mTORC1 Reactivation During Amino Acid Starvation,” Nature Communications 8, no. 1 (2017): 338. 10.1038/s41467-017-00369-y PMC5569045 28835610

[32] Y. Sancak , L. Bar‐Peled , R. Zoncu , A. L. Markhard , S. Nada , and D. M. Sabatini , “Ragulator‐Rag Complex Targets mTORC1 to the Lysosomal Surface and Is Necessary for Its Activation by Amino Acids,” Cell 141, no. 2 (2010): 290–303. 20381137 10.1016/j.cell.2010.02.024 PMC3024592

[33] O. M. Kolaczkowski , B. A. Goodson , V. M. Vazquez , J. Jia , A. Q. Bhat , T. H. Kim , and J. Pu , “Synergistic Role of Amino Acids in Enhancing mTOR Activation Through Lysosome Positioning,” bioRxiv (2024). 2024.10.12.618047. 10.1101/2024.10.12.618047.

[34] R. M. Bocek , R. D. Peterson , and C. H. Beatty , “Glycogen Metabolism in red and White Muscle,” American Journal of Physiology 210, no. 5 (1966): 1101–1107. 5947256 10.1152/ajplegacy.1966.210.5.1101

[35] S. Pickles , P. Vigie , and R. J. Youle , “Mitophagy and Quality Control Mechanisms in Mitochondrial Maintenance,” Current Biology 28, no. 4 (2018): R170–R185. 29462587 10.1016/j.cub.2018.01.004 PMC7255410

[36] R. J. Braun , C. Sommer , C. Leibiger , et al., “Accumulation of Basic Amino Acids at Mitochondria Dictates the Cytotoxicity of Aberrant Ubiquitin,” Cell Reports 10, no. 9 (2015): 1557–1571. 25753421 10.1016/j.celrep.2015.02.009 PMC4407011

[37] C. E. Hughes , T. K. Coody , M. Y. Jeong , J. A. Berg , D. R. Winge , and A. L. Hughes , “Cysteine Toxicity Drives Age‐Related Mitochondrial Decline by Altering Iron Homeostasis,” Cell 180, no. 2 (2020): 296–310 e18. 31978346 10.1016/j.cell.2019.12.035 PMC7164368

[38] M. H. Schuler , A. M. English , T. Xiao , T. J. Campbell , J. M. Shaw , and A. L. Hughes , “Mitochondrial‐Derived Compartments Facilitate Cellular Adaptation to Amino Acid Stress,” Molecular Cell 81, no. 18 (2021): 3786–3802 e13. 34547239 10.1016/j.molcel.2021.08.021 PMC8513802

[39] M. Wiessner , A. Roos , C. J. Munn , et al., “Mutations in INPP5K, Encoding a Phosphoinositide 5‐Phosphatase, Cause Congenital Muscular Dystrophy With Cataracts and Mild Cognitive Impairment,” American Journal of Human Genetics 100, no. 3 (2017): 523–536. 28190456 10.1016/j.ajhg.2017.01.024 PMC5339217

[40] D. P. S. Osborn , H. L. Pond , N. Mazaheri , et al., “Mutations in INPP5K Cause a Form of Congenital Muscular Dystrophy Overlapping Marinesco‐Sjogren Syndrome and Dystroglycanopathy,” American Journal of Human Genetics 100, no. 3 (2017): 537–545. 28190459 10.1016/j.ajhg.2017.01.019 PMC5339112

[41] M. J. McGrath , M. J. Eramo , R. Gurung , et al., “Defective Lysosome Reformation During Autophagy Causes Skeletal Muscle Disease,” Journal of Clinical Investigation 131, no. 1 (2021): e135124. 33119550 10.1172/JCI135124 PMC7773396

[42] R. R. Pitere , M. B. van Heerden , M. S. Pepper , and M. A. Ambele , “Slc7a8 Deletion Is Protective Against Diet‐Induced Obesity and Attenuates Lipid Accumulation in Multiple Organs,” Biology (Basel) 11, no. 2 (2022): 311. 35205177 10.3390/biology11020311 PMC8869389

