# Correction to ‘Ablation of LAT2 Transporter Causes Intramuscular Glutamine Accumulation and Inhibition of Fasting‐Induced Proteolysis’

**DOI:** 10.1002/jcsm.70015

**Published:** 2025-07-27

Espino‐Guarch, M. , Huang, S. , Vilches, C. , Prat, E. , El Nahas, R. , Missous, G. , Bodoy, S. , Sathappan, A. , Al‐Aghbar, M. , Mayayo, C. , Olivé, M. , Busquets‐Rius, S. , Sebastián, D. , Zorzano, A. , Palacin, M. , van Panhuys, N. and Nunes, V. (2025), Ablation of LAT2 Transporter Causes Intramuscular Glutamine Accumulation and Inhibition of Fasting‐Induced Proteolysis. Journal of Cachexia, Sarcopenia and Muscle, 16: e13847, 10.1002/jcsm.13847.40546137 PMC12183528

The following Acknowledgment section is missing:

Open Access funding provided by the Qatar National Library.

We apologize for this error.

