# Supplementary material for: Ablation of LAT2 Transporter Causes Intramuscular Glutamine Accumulation and Inhibition of Fasting‐Induced Proteolysis
Source: J Cachexia Sarcopenia Muscle. 2025 Jun 23;16(3):e13847. doi: 10.1002/jcsm.13847 (PMC12183528; doi:10.1002/jcsm.13847)
Supplement: Supplementary file 1 — Figure S1. RNA‐Seq data analysis. (A) Venn diagram representing DEG’s for each comparison. (B) Heat map demonstrating clustering of top 2000 most variable up‐ and down‐regulated genes for individual animals from WT and LAT2KO in fed and 48‐h fasted groups. (C) PCA plot indicating principal components that efficiently segregate fasting (PC1) and genotype (PC4). Figure S2. Absolute tissue weight of fed and 16‐ and 48‐h fasted mice. Mean + SEM of absolute tissue weight of liver, kidneys, pancreas, spleen, and brain from 6 to 8 mice. Mean + SEM of relative tissue weight to mice total weight of gastrocnemius, quadriceps, and abdominal adipose tissue (WAT) from 6 to 8 mice. Wild type (orange) and LAT2 knockout (turquoise) samples are presented. Wilcoxon rank sum test p values are represented by *< 0.05 and **< 0.01. Figure S3. Effects of LAT2 knockout on muscle morphology and function. (A) Histopathology of WT and LAT2KO gastrocnemius muscle sections. Haematoxylin and eosin (H/E), modified Gömori trichrome (mGT), Periodic acid Schiff technique (PAS), reduced nicotinamide adenine dinucleotide dehydrogenase‐tetrazolium reductase (NADH), cytochrome c oxidase (COX), and succinic dehydrogenase (SDH) were used to evaluate muscle tissue from fed animals (N = 8 per genotype). Scale bar represents 50 mm for H/E, PAS, NADH and SDH; 20 mm for mGT and 100 mm for COX staining. (B) Cross sectional area (CSA) distribution and means (dots represent each quantified area) of fed gastrocnemius muscle (N = 6 per genotype). (C) Grip strength test results showing minimum (min) and maximum (max) strength recorded per animal and assessed in triplicate. (D) Results of treadmill increasing speed test performed for a fixed distance and after training session (N = 12 mice per genotype). Number of stops represent each time the mice felt from the running belt into the shock chamber. (E) Mitochondrial copy numbers measured by real time PCR. Median and percentiles are presented (N = 12 per genotype). (F [file JCSM-16-e13847-s001.docx]

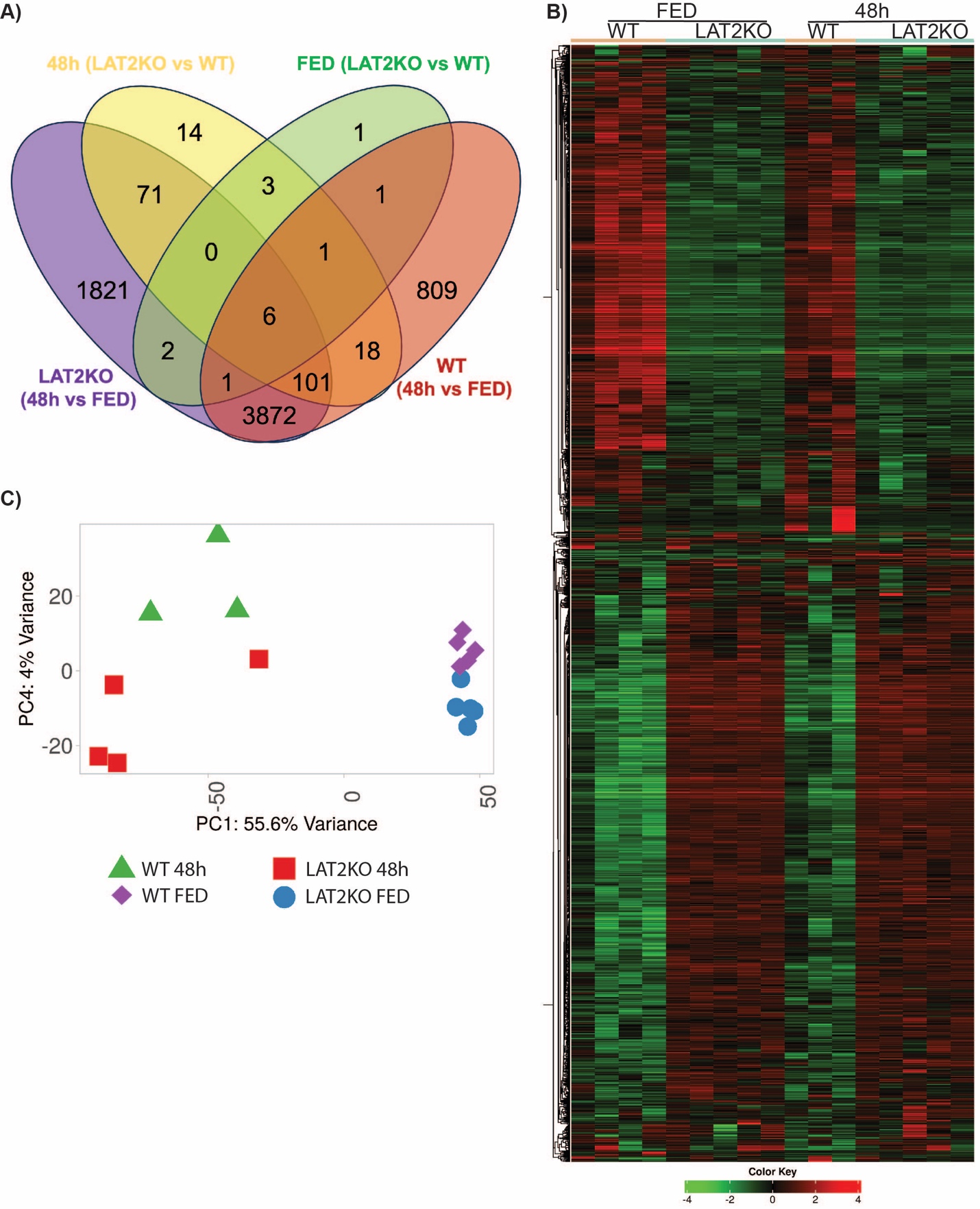


**Supplementary Figure S1: RNA-Seq data analysis. (A)** Venn diagram representing DEG’s for each comparison. **(B)** Heat map demonstrating clustering of top 2000 most variable up- and down-regulated genes for individual animals from WT and LAT2KO in fed and 48-h fasted groups. **(C)** PCA plot indicating principal components that efficiently segregate fasting (PC1) and genotype (PC4).

**
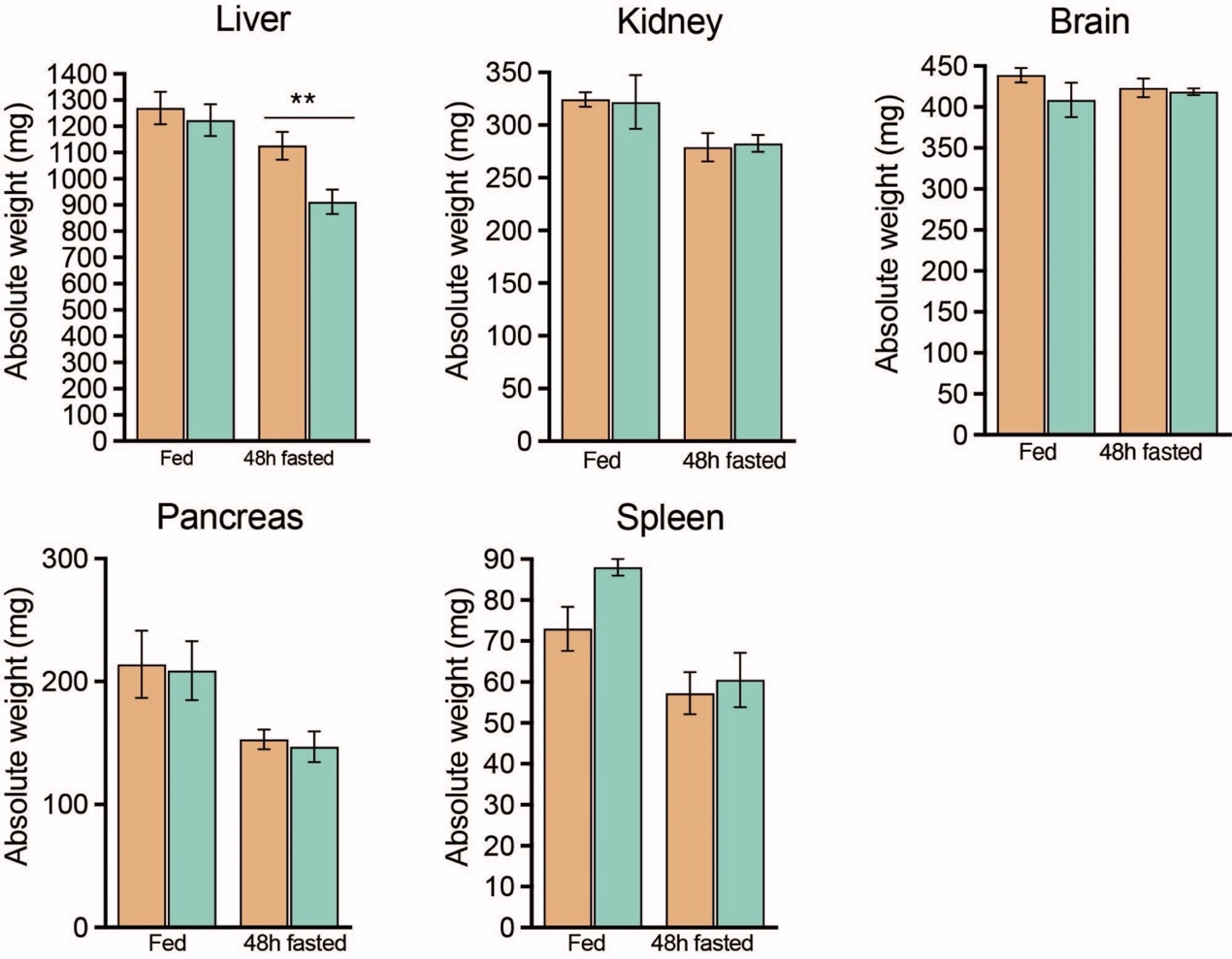
**

**Supplementary Figure S2: Absolute tissue weight of Fed and 16-h and 48-h fasted mice** Mean + SEM of absolute tissue weight of liver, kidneys, pancreas, spleen, and brain from 6-8 mice. Mean + SEM of relative tissue weight to mice total weight of gastrocnemius, quadriceps, and abdominal adipose tissue (WAT) from 6-8 mice. Wild type (orange) and LAT2 knockout (turquoise) samples are presented. Wilcoxon rank sum test p-values are represented by *<0.05 and **<0.01.

**
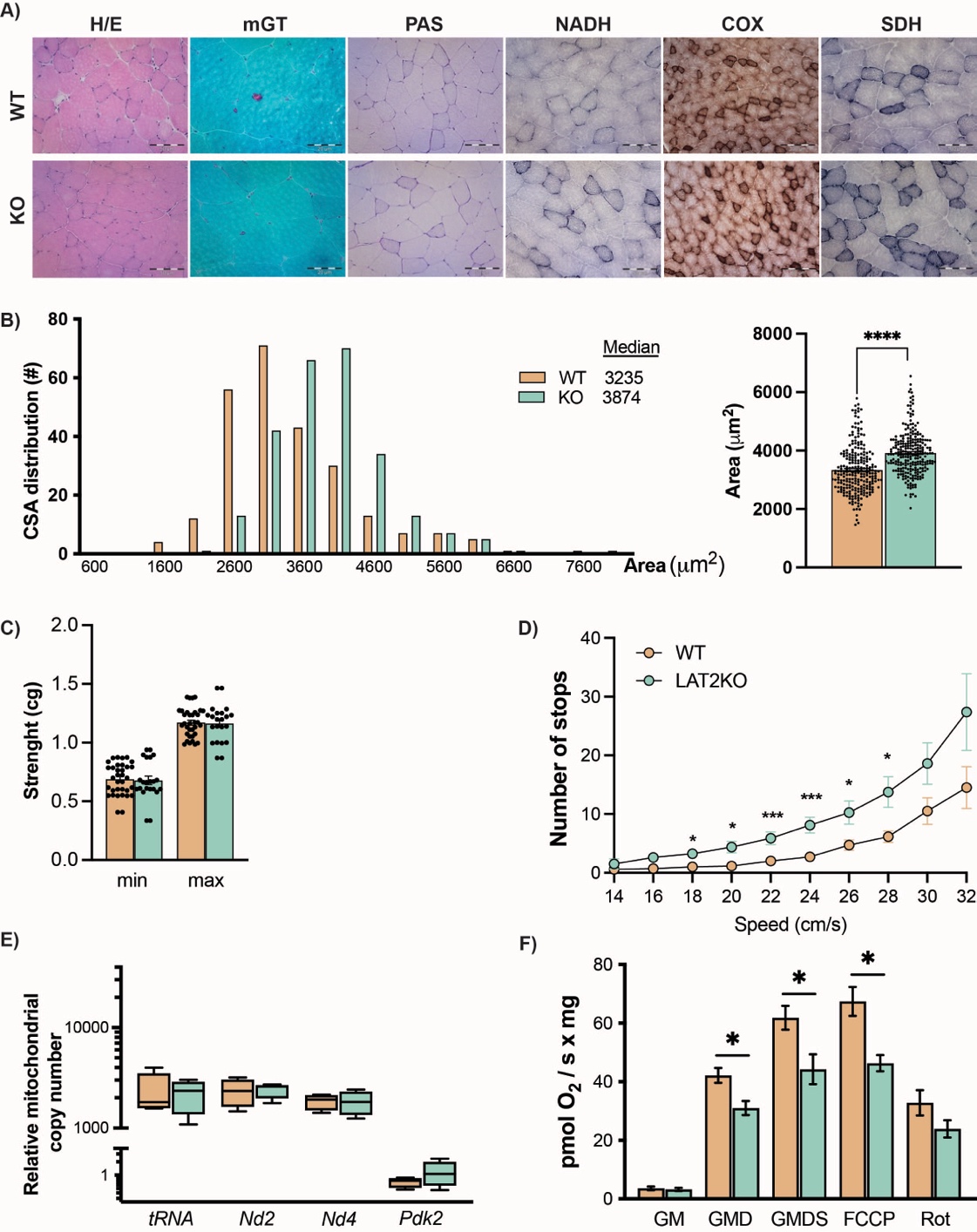
**

**Supplementary Figure S3. Effects of LAT2 knockout on muscle morphology and function. (A)** Histopathology of WT and LAT2KO gastrocnemius muscle sections. Hematoxylin and eosin (H/E), modified Gömori trichrome (mGT), Periodic acid Schiff technique (PAS), reduced nicotinamide adenine dinucleotide dehydrogenase-tetrazolium reductase (NADH), cytochrome c oxidase (COX), and succinic dehydrogenase (SDH) were used to evaluate muscle tissue from fed animals (N = 8 per genotype). Scale bar represents 50mm for H/E, PAS, NADH and SDH; 20mm for mGT and 100mm for COX staining. **(B)** Cross sectional area (CSA) distribution and means (dots represent each quantified area) of fed gastrocnemius muscle (N = 6 per genotype). **(C)** Grip strength test results showing minimum (min) and maximum (max) strength recorded per animal and assessed in triplicate. **(D)** Results of treadmill increasing speed test performed for a fixed distance and after training session (N = 12 mice per genotype). Number of stops represent each time the mice felt from the running belt into the shock chamber. **(E)** Mitochondrial copy numbers measured by real time PCR. Median and percentiles are presented (N = 12 per genotype). **(F)** Oxygen composition analysis by high-resolution respirometry of permeabilized muscle according to tissue weight. GM: glutamate + pyruvate (basal), GMD: addition of ATP (CI), GMDS: addition of succinate (CII), FCCP: Addition of uncouplers (CI+CII max), and Rot: addition of rotenone inhibitor (CII max). **(B-F)** Mean + SEM of WT (orange) and LAT2 knockout (KO) (turquoise) samples are presented. Wilcoxon rank sum test p-values are represented by *<0.05, ***<0.001 and ****<0.0001.


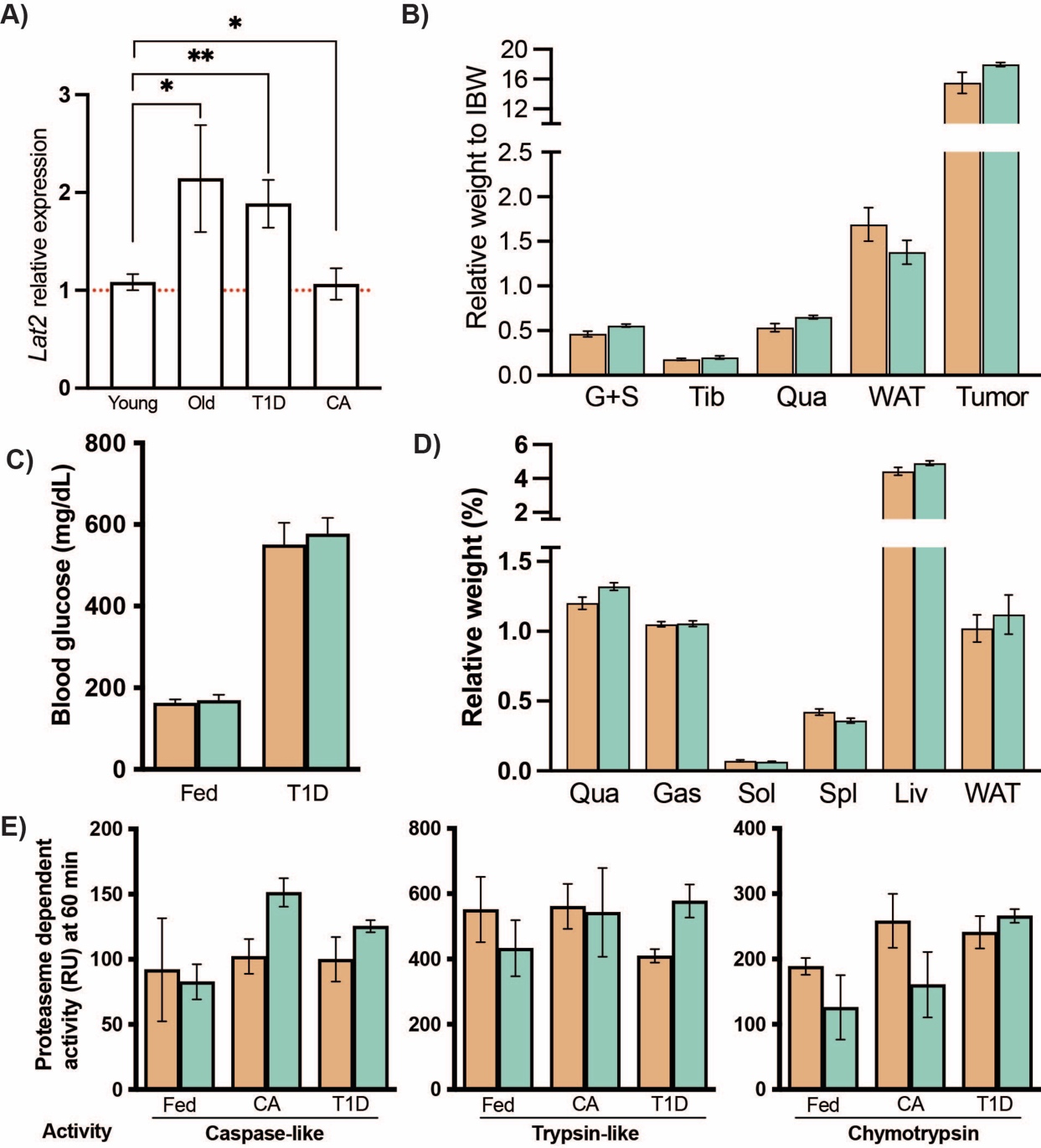


**Supplementary Figure S4: Effect of LAT2 knockout on chronic diseases with increased proteolysis (A)** Analysis of *Lat2* expression by real time PCR in muscle from aged, type 1 diabetes (T1D), Lewis’s cancer-cachexia (CA), and young wild type (WT) mice normalized to GAPDH. **(B)** Relative tissue weight normalized to initial body weight (IBW) of CA model for gastrocnemius + soleus (G+S), tibialis (Tib), quadriceps (Qua), abdominal white adipose tissue (WAT), and tumor of 6 mice per genotype injected with CA cell suspension. **(C)** Blood glucose levels in Fed and streptozotocin-induced T1D mice. **(D)** Relative tissue weight normalized to body weight of T1D model for Qua, gastrocnemius (Gas), soleus (Sol), spleen (Spl), Liver (Liv), and WAT of 8 T1D mice per genotype. **(E)** Gastrocnemius muscle proteasome activity measurements by fluorometric assay of Fed, CA, and T1D models (N = 6 per genotype and condition)**. (B-E)** WT (orange) and LAT2 knockout (KO) (turquoise) samples are presented. Wilcoxon rank sum test p-values are represented by *<0.05 and **<0.01.

**Supplementary Table S1: AA content of skeletal muscle** AA content of G+S muscles in Fed and 16-h and 48-h fasted mice, per mg of tissue (N = 6-12 per condition and genotype); Mean + SEM and ratios (fold change, LAT2KO vs WT) are represented. Wilcoxon rank sum test p-values are represented by *<0.05, **<0.01, and ***<0.001.


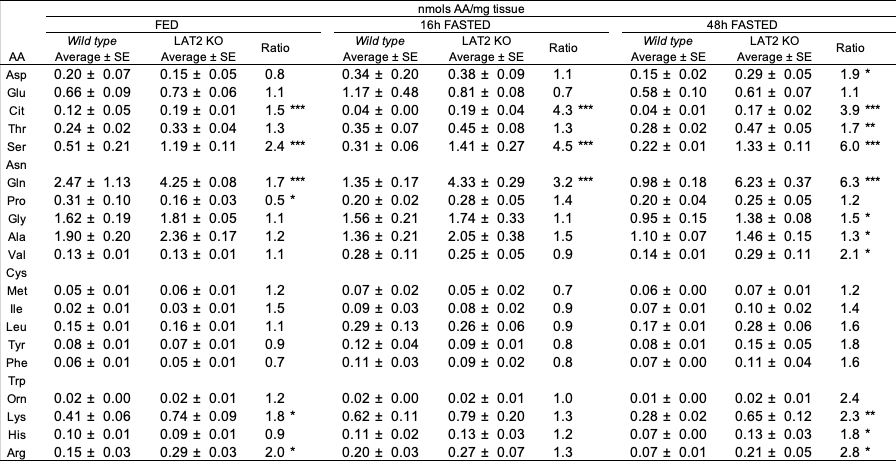


**Supplementary Table S2: AA content of liver** AA content of liver in Fed and 16-h fasted mice, per mg of tissue (N = 6-12 per condition and genotype); mean + SEM and ratios (fold change, LAT2KO vs WT) are presented. Wilcoxon rank sum test p-values are represented by *<0.05 and **<0.01.


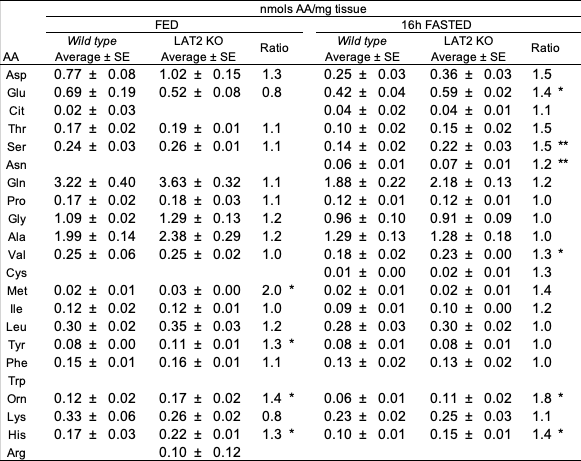


**Supplementary Table S3: Amino acid content of blood** Plasma amino acid (AA) content in Fed and 16-h and 48-h fasted mice, according to volume of plasma (N = 6-12 per condition and genotype); mean + SEM and ratios (fold change, LAT2KO vs WT) are presented. Wilcoxon rank sum test p-values are represented by *<0.05 and ***<0.001.


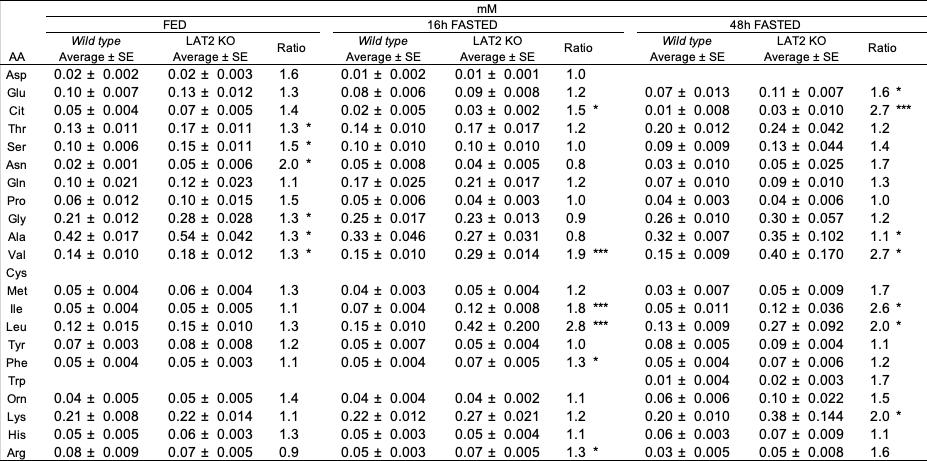


**Supplementary Table S4: Gene expression of amino acid transporters in G+S muscle** Gene expression of Fed and 16-h fasted (FAST) WT and LAT2KO mice, analyzed using Roche UPL-System (N = 6 per condition and genotype). Substrate: AAs are shown as single-letter codes, AA0: neutral amino acids. Mean + SEM of fold change (LAT2KO vs WT) is presented. Wilcoxon rank sum test p-values are represented by *<0.05, **<0.01, and ***<0.001.

**
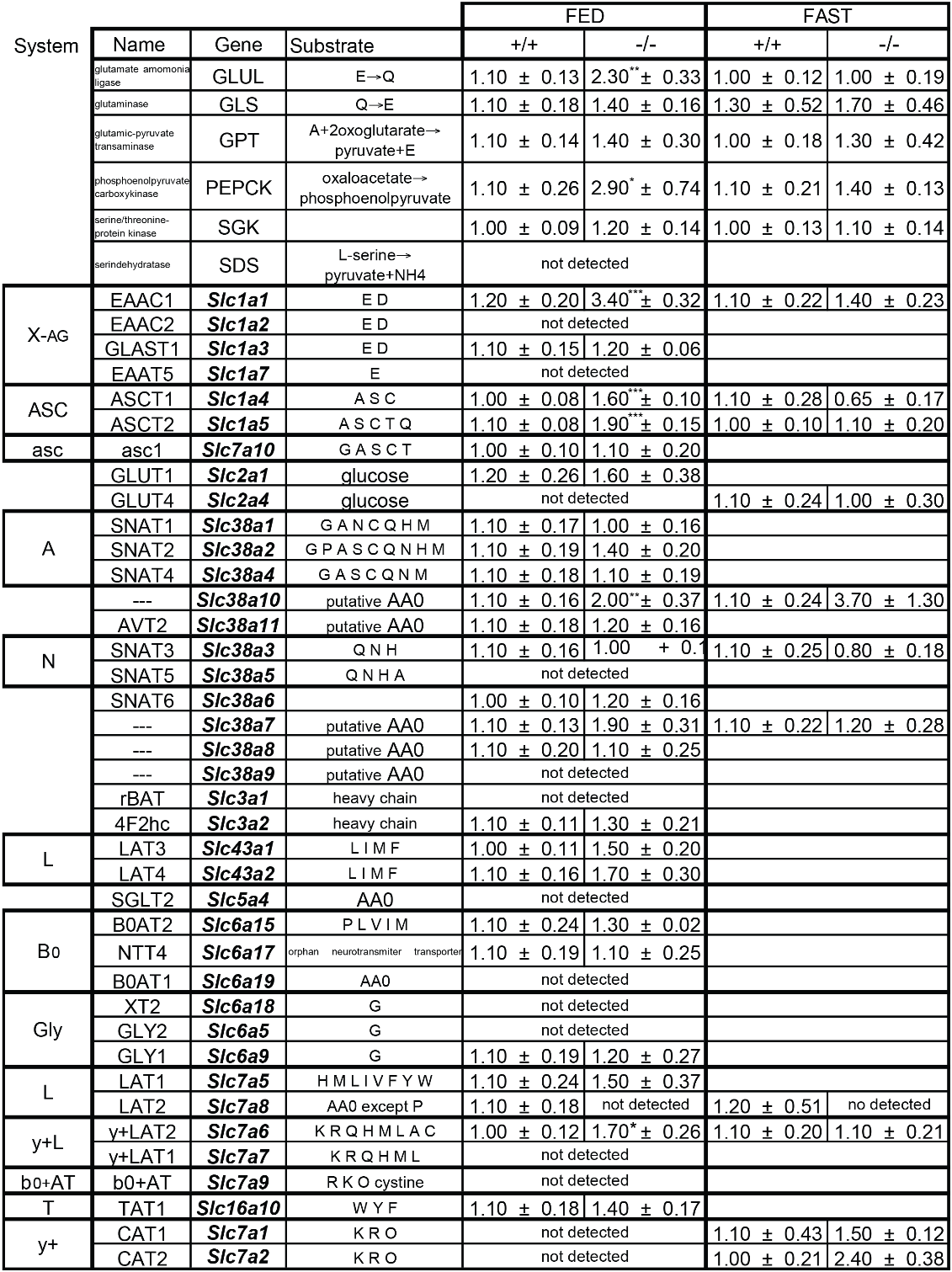
**

**Supplementary Table S5: Methodology details. (A)** Primers used for real-time PCR. Symbol of the gene name. Ref. for mRNA gene sequence. Primer’s sequence shown as 5’ to 3’. Roche UPL probe identifier and amplicon size. **(B)** Antibodies used for western blot and immunofluorescence. Protein name. Reference for commercial details (catalog number, Company). WB dil indicates dilutions and buffer used for western blot. IF dil indicates dilution used for immunofluorescence and (if) secondary antibody (Ab) used and its dilution (Dil sec).

**
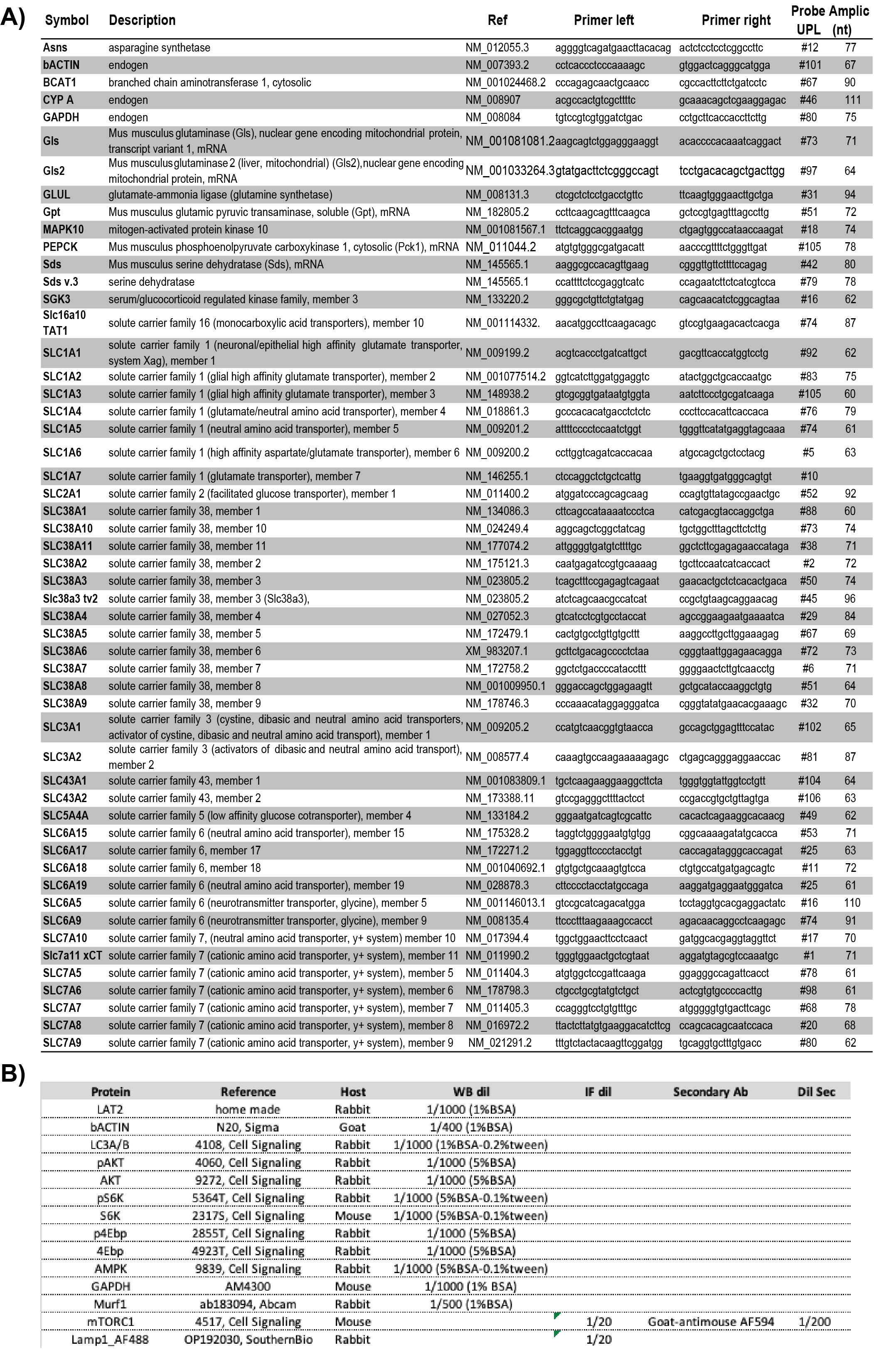
**
